# Work above shoulder level and shoulder complaints: a systematic review

**DOI:** 10.1007/s00420-020-01551-4

**Published:** 2020-06-22

**Authors:** Morten Wærsted, Markus Koch, Kaj Bo Veiersted

**Affiliations:** National Institute of Occupational Health, Gydas vei 8, Majorstuen, PO Box 5330, 0304 Oslo, Norway

**Keywords:** Systematic review, Work-related musculoskeletal disorders, Shoulder pain, Arm elevation

## Abstract

**Objective:**

To investigate the association and the exposure–response relationship between work above shoulder height and shoulder pain or disorders.

**Methods:**

A systematic search was performed in Medline, Embase, and Health and Safety Science Abstracts. Included were articles with prospective cohort, case–control, cross-sectional, or intervention study designs. Quality assessment was based on an evaluation scheme adjusted to study design and normalized to 100%. The cut-off for sufficient quality to include articles was above 40% and cut-off for high-quality articles was above 50% of maximal score. The level of strength of evidence for an association between exposure and effect was assessed according to the GRADE guidelines.

**Results:**

Thirty-four articles were included. Articles that document large effects (higher risk estimates; OR ≥ 2) have higher quality score, include analyses of severe arm elevation, more often use clinical outcome, and report an exposure–response relationship compared to studies reporting lower risk estimates. The studies that reported large effects were all significant.

An exposure–response relationship was found in many high-quality studies when relating exposure intensity of arm elevation (level of arm elevation, amplitude) as well as duration of arm elevation, especially > 90°.

**Conclusion:**

We conclude on a limited evidence for an association between arm elevation at work and shoulder disorders. Severe arm elevation with elbows above shoulder level (i.e., > 90°) shows a moderate evidence for an association with shoulder disorders.

**Electronic supplementary material:**

The online version of this article (10.1007/s00420-020-01551-4) contains supplementary material, which is available to authorized users.

## Introduction

Shoulder pain or disorders are a widespread in the general population. In a systematic review, Luime and co-workers found prevalence rates for 1-month prevalence of shoulder pain ranging from 19 to 31%, 5–47% for 1-year prevalence, and 7–67% for lifetime prevalence (Luime et al. 2004a). In a study conducted in Sweden, the estimated costs per patient seeking primary health care with shoulder pain were in average €326 for healthcare and €1743 for sick leave during a period of 6 months (Virta et al. 2012). For patients with higher need for medical care, the total costs increased dramatically (€8528). With a focus on the high socioeconomic burden and individual’s health and work ability, a reduction in occurrence and severity of musculoskeletal disorders are wanted.

Previous reviews have shown a positive association between work with hands above the shoulder and shoulder disorders (Mayer et al. 2012; van der Molen et al. 2017). Still, the studies reviewed by Mayer and co-workers showed no statistically significant associations (Mayer et al. 2012). Other reviews examined only the association of work above shoulder level with the combined outcome neck-shoulder pain (Larsson et al. 2007), referred only a few studies (Sommerich et al. 1993) or included work above shoulder level in some other categories of risk factors, making it difficult to give a clear statement on the associations.

“Work above shoulder level” is conceptually a vague description of exposure, which includes postures with very different load on the shoulder structures and presupposes the torso in an upright position. Previous studies have many expressions of this kind of exposure; ‘work above shoulder height’ (Mikkonen et al. 2012), ‘hands above shoulder height’ (Wiktorin et al. 1999), ‘overhead work’ (Herberts et al. 1981; Sakakibara et al. 1987; Tanii et al. 1972), and ‘arms above shoulder level’. In the present review, we included all these mentioned terms.

Technical advancements allow a more accurate examination of work exposures, e.g., by wearable inclinometers. The exposure can be measured during the whole working day, in leisure time, and even over several subsequent days. Compared to the participants’ subjective estimates in questionnaires, the measured exposure durations are smaller, meaning that participants have a tendency to overestimate the duration of work above shoulder height (Koch et al. 2016). Receiving more accurate exposure measurements might, therefore, lead to modified associations of work above shoulder level with shoulder pain or disorders. However, most of the scientific evidence available at present do not include technical measurements, and the articles with technical measurements or exposure assessment by video recordings include on average relatively few participants due to high demands on resources for data collection and technical expertise.

The mechanisms for the pathophysiology, relating arm elevation at work to reduced musculoskeletal health have been widely discussed; however, no consensus exists. Besides, possibly several of the proposed mechanism may play a part. Muscular fatigue (Armstrong et al. 1993; Kumar 2001), prolonged muscle activation (Hägg 1991; Visser and van Dieën 2006), cumulative trauma disorder (Kumar 2001), inflammatory processes (Barbe and Barr 2006), reduced microcirculation (Palmerud et al. 2000; Visser and van Dieën 2006), and mechanical static or repetitive pressure on the tendons (Seitz et al. 2011) are all suggested as possible mechanisms. A pressure on the rotator cuff tendons by the undersurface of acromion occurs when arms are elevated, especially between 60° and 120° (Levitz and Iannotti 1995).

The present review investigates the association and the exposure–response relationship between work above shoulder height and shoulder pain or disorders. To our knowledge, a systematic critical review focusing only on arm elevation at work as a possible risk factor has not been performed previously.

## Methods

### Literature search

A systematic search for scientific literature published from 01.01.1990 until 01.03.2018 was performed in the databases Medline® (US National Library of Medicine, Bethesda, United States), Embase® (Elsevier Limited, Oxford, UK), and Health and Safety Science Abstracts (Rutgers, The State University of New Jersey). We used a term bundle of Medical Subject Headings (MesH) based on 24 articles that by our experiences should be included (Table 1). Appendix 1 lists in detail the search strategy used in the three databases. After removing duplicates, the results were merged into one EndNote database (EndNoteX8.0.2, PDF Tron TM Systems Inc., Vancouver, Canada).

**Table 1:** Included MesH terms for the literature search

Exposures	Outcome
Arm elevation Arms above shoulder Elbow above shoulder Employment Hands above shoulder Occupational disease Occupational exposure Occupations Overhead work Shoulder load Shoulder muscular load Work Work above shoulder heights Workload Workplace Work-related	Acromioclavicular joint Adhesive capsulitis Arthrosis Bicipital tendinitis Bursitis Capsulitis Degenerative arthritis Frozen shoulder Glenohumeral arthrosis Joint disease Joint instability Movement Myofascial pain syndromes Osteoarthritis Rotator cuff Rotator cuff syndrome Rotator cuff tear Shoulder Impingement Syndrome Shoulder adhesive capsulitis Shoulder complain Shoulder dislocation Shoulder disorder Shoulder joint Shoulder pain Shoulder tendinitis Subacromial impingement syndrome Tendinopathy Trigger points

**Fig. 1:**
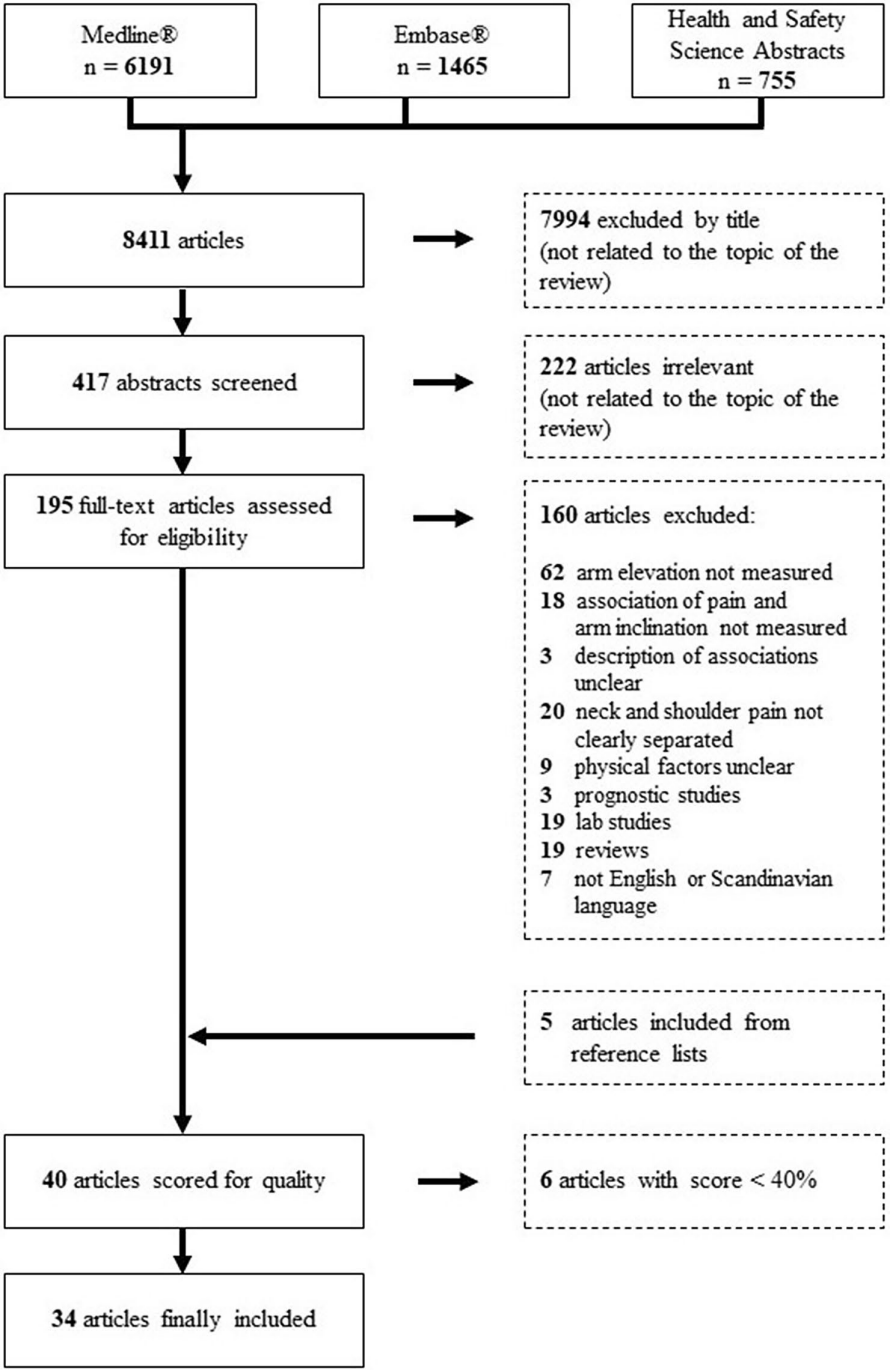
Flowchart of the literature review. The figure shows the review process from database screening to the finally included 34 articles

We found 6191 articles in the Medline database plus 1465 new articles from the Embase database and 755 from Health and Scientific Abstracts. One author (MK) checked the collected 8411 articles by title (7994 articles excluded), abstract (222 articles excluded), and finally full-text (160 studies excluded, see Appendix 2) for the inclusion and exclusion criteria. To increase the number of relevant studies included, the reference lists of the most recent included studies (Bovenzi 2015; Coenen et al. 2016; Dalbøge et al. 2018a; Hanvold et al. 2015; Koch et al. 2017; Nordander et al. 2016), as well as two recent reviews (Dalbøge et al. 2018b; van der Molen et al. 2017), were checked and five extra studies were included. Figure 1 presents a flowchart of the article selection process.

### Inclusion criteria

#### Work-related studies

The included articles had to investigate exposures during working time. This included work in various professions or with various working tasks.

#### Exposures

We only included articles specifying relevant exposures during working time, e.g., overhead work, work with elevated arms, hands above shoulder height, arms above shoulder level or studies quantifying arm inclination by video, or inclinometry.

#### Outcomes

The included articles had to investigate the associations with pain, discomfort, clinical signs, or clinical diagnoses in the shoulder region.

#### Study design

In this review, we included all epidemiological study designs (case–control, cross-sectional, intervention, and prospective cohort studies). This included register and population studies.

### Exclusion criteria

We excluded:

- Results on the outcome neck and shoulder pain (not separated).
- Results on sickness absence or disability pensioning.
- Results of exposures due to war or acts of terrorism.
- Studies on athletes (also professional athletes).
- Intervention studies that did not specifically deal with work-related interventions.
- Results concerning patients with cancer or diabetes.
- Evaluations of treatment for shoulder pain (prognostic studies).

### Quality control

The included 40 articles were quality controlled independently by two of the authors. Colleagues at the institute evaluated included articles by authors of this report. The quality control was performed with a scoring scheme (Appendix 3) which we have used in the previous reviews (Knardahl et al. 2008, 2017; Veiersted et al. 2017). The scoring schemes differed slightly for quality assessment of prospective cohort, case–control, intervention, and cross-sectional studies. The quality score was normalized to a maximum of 100%. Agreement between the two reviewers was checked for all items. When different, the two reviewers agreed on a common score for the specific items by assessing the article together. We decided to include all articles receiving a quality score of more than 40%, designating articles with quality rating > 50% as high quality. Six articles were excluded due to low-quality score (Dainty et al. 2014; Northover et al. 2007; Oliveira Dantas and de Lima 2015; Sakakibara et al. 1995; Seaman et al. 2010; Thetkathuek et al. 2017). Two of the authors (MK and MW) extracted independently the results from the included articles. Presentation of the report is to a large extent according to the PRISMA statement from 2009 (Liberati et al. 2009).

### Establishing strength of evidence

We have used the GRADE method (Grading of Recommendation Assessment, Development, and Evaluation) (Guyatt et al. 2011a) to summarize and discuss the evidence for the strength of a correlation between a specific occupational exposure factor and various musculoskeletal disorders. A minor modification of the method was implemented, as it was done in an earlier report from the Swedish Council on Health Technology Assessment (SBU 2014): If one finds great consistency between several studies with good handling of sources of error, evidence strength can be increased by one level. Meta-analyses, forest plots, and, e.g., calculations of publication bias by funnel plots were not used due the relative heterogenic exposure assessments and outcome measures.

The strength of evidence of relationship between exposure and effect in observation studies is graded in four levels. The higher the evidence strength, the greater the likelihood that the results are stable over time and will not change with new research. Also limited evidence strength means that there is scientific basis for the existence of a correlation (sufficient evidence), but this connection is uncertain and can be changed in future research.

*Strong evidence (‡‡‡‡)* The scientific basis consists of randomized studies without bias of significance. There is little likelihood that the conclusion will change in future research.

*Moderate evidence (‡‡‡)* The scientific basis consists of high- or intermediate-quality observation studies for which reinforcing conditions exist. There is a moderate likelihood that the conclusion can be changed in future research.

*Limited evidence (‡‡)* The scientific basis consists of high- or intermediate-quality observation studies. There is a greater likelihood that the conclusion may be changed by future research, but there is still sufficient evidence for a coherence.

*Insufficient evidence (‡)* Lack of scientific basis, either in the number of studies or in the absence of good quality. A weakened strength of evidence may occur if sufficient quality observational studies have inconsistent results, or only one single high-quality study was found (Guyatt et al. 2011a). It is possible that the conclusion can be changed in future research.

Table 2 summarizes the modified GRADE method used in this review.

**Table 2:** Assessment of evidence in relation to modified GRADE method

Strength of evidence	Symbol	Study design
Strong	‡‡‡‡	Randomized studies
Moderate	‡‡‡	
Limited	‡‡	Observational studies
Insufficient	‡	Case–control studies only

Strength reduced with weakening conditions		Strength increased with reinforcing conditions	
Flaws in study quality	Max-2	Large effects and few confounders	Max + 2
Low consistency between studies	Max-2	High compliance between studies*, and good handling of confounders**	Max + 1
Lack of transferability or relevance	Max-2	Clear dose–response relationship, or altered exposure gives change in effect	Max + 1
Low precision	Max-2	Confounders that are not included in analysis are likely to underestimate context	Max + 1
High risk of publication bias	Max-2		

Modified after Balshem et al. (2011), Guyatt et al. (2011a, b, c, d), and SBU (2014)

*Additional criteria to increase the strength one level: will affect a larger group of people in their usual environment/work; quality assessment before evidence assessment; only high- or intermediate-grade studies can be used; multiple studies with heterogeneous populations

**Confounders are taken care of by adjusting or by study design

## Results

Table 3 gives an overview of the 34 included articles, with information on design, quality score, general method for the description of exposure and outcome, number of study participants, and their occupational background if known. Appendix 4 lists the confounder variables included in the analyses. The specific results from each article are summarized in four tables depending on the method for exposure assessment; by questionnaire or interview (20 studies, Table 4), expert rating (4 studies, Table 5), video observation (5 studies, Table 6), or inclinometry (5 studies, Table 7). The order of exposure assessment methods was decided by assumed an increasing level of validity and precision. The study outcomes were defined as pain, discomfort or complaints, during last week, month, past 6 months or 1 year, or medical diagnoses based on clinical examinations only or, e.g., time of first impingement syndrome surgery. The clinical diagnosed disorders in included articles were rotator cuff syndrome, subacromial impingement syndrome, partial or total supraspinatus tendon tears, supraspinatus tendinopathy, and AC joint degeneration. Except for the last diagnosis, the other shoulder diagnoses may be pooled together as one entity, rotator cuff disorders. To what extent, the articles adjusted for confounders in the multivariate analyses varied. All articles controlled for age and gender, either by design or in the analyses. One article did only adjust for age and gender (Nahit et al. 2001), three articles adjusted for one extra confounder [BMI (Silverstein et al. 2008, 2009) or pain in other sites (Hoe et al. 2012)], while the remaining articles adjusted for several confounders in addition to age and gender in their analyses. These confounders included other work-related risk factors and/or individual risk factors for shoulder disorders. Appendix 4 gives an overview of the confounders adjusted for in the included articles.

**Table 3:** Overview of included articles: alphabetic order

	Study design	Qual score	Assessment outcome	Exposure	*N*	Professions
Bodin (2012a)	CS	58	Questionnaire Physical examination	Questionnaire	3710	Various
Bodin (2012b)	PC	51	Physical examination	Questionnaire	3710	Various
Bodin (2012c)	PC	53	Questionnaire	Questionnaire	1655	Various
Bovenzi (2015)	PC	72	Questionnaire Numerical rating scale	Questionnaire Interview	537	Drivers
Coenen (2016)	PC	63	Questionnaire	Questionnaire Video observation	789 (video 245)	Various
Dalbøge (2018a)	PC	63	Date of surgery Questionnaire	JEM based on expert ratings + inclinometer	2,374,403 (Inclin. 575)	Various
Dalbøge (2017)	CC	76	First-time surgery Questionnaire	JEM based on expert ratings + inclinometer	5396	Various
Dalbøge (2014)	PC	58	Date of surgery Questionnaire	Expert ratings	2,374,403	Various
Descatha (2012)	PC	46	Questionnaire	Questionnaire	1786	Various
Engholm (2005)	CS	47	Questionnaire	Questionnaire	85,191	Construction sector
Hanvold (2015)	PC	67	Questionnaire Pain drawing	Inclinometer	41	Hairdressers Electricians, Students Various
Harkness (2003)	PC	65	Questionnaire Pain drawing	Questionnaire	1081	Various
Hoe (2012)	CS	47	Questionnaire	Questionnaire	1111	Nurses
Hooftman (2009)	PC	67	Questionnaire	Questionnaire	1789	No information
Hoozemans (2002)	CS	53	Questionnaire	Questionnaire	622	Pushing pulling professions
Koch (2017)	PC	56	Questionnaire	Inclinometer Questionnaire	125	Construction and health care workers
Leclerc (2004)	PC	44	Questionnaire	Questionnaire	598	Various
Luime (2004b)	PC	47	Questionnaire	Questionnaire	769	Nursing-home and elderly care workers
Melchior (2006)	CS	53	Questionnaire Physical examination	Questionnaire	2656	Various
Miranda (2005)	CS	78	Questionnaire Interview Physical examination	Questionnaire Interview	4071	Various
Miranda (2001)	PC	44	Questionnaire	Questionnaire	3312	Various
Nahit (2001)	CS	44	Pain drawing	Questionnaire	1081	Various
Niedhammer (1998)	CS	44	Questionnaire Pain drawing	Questionnaire	210	Supermarket cashiers
Nordander (2016)	CS	58	Questionnaire Physical examination	Inclinometer	3141	Various
Punnett (2000)	CC	73	Questionnaire Physical examination	Video recordings	79/124 (cases/cont)	Automobile assembly workers
Roquelaure (2011)	CS	58	Questionnaire Physical examination	Questionnaire	3710	Various
Seidler (2011)	CC	57	Clinical diagnosis MRI	Interview	483/300 (cases/cont)	Various
Silverstein (2009)	CS	62	Questionnaire Interview Physical examination	Video observation	733	Manufacturing and health care workers
Silverstein (2008)	CS	64	Questionnaire Interview Physical examination	Video observation	733	Manufacturing and health care workers
Sim (2006)	CS	44	Pain drawing	Questionnaire	5133	Various
Smith (2009)	PC	67	Questionnaire	Video recordings	424	Various
Svendsen (2013)	PC	53	Date of surgery	Expert ratings	37,402	Various
Svendsen (2004a)	CS	75	Questionnaire Physical examination	Questionnaire Inclinometer	1886 (Inclin. 72)	Male machinists, car mechanics, house painters
Svendsen (2004b)	CS	67	MRI	Inclinometer	136	Male machinists, car mechanics, house painters

Study design: *PC* prospective cohort, *CC* case–control, *CS* cross-sectional

*JEM* job-exposure matrix

**Table 4:** Results from studies using questionnaires or interviews to assess work with elevated arms (self-reported): alphabetic order

	Outcome	Exposure	OR (95% CI)	
			Univariate	Multivariate
Bodin (2012a) CS *Q* = 58 clinical assessment	Shoulder pain during in the past 12 months with/ without rotator cuff syndrome (RCS)	Working with arms abducted > 60° and/or above shoulder level ≥ 2 h/day	Not available	Shoulder pain without RCS: Men Exposed < 2 h: 1.0 (reference) Abducted: 1.6 (1.2; 2.2) Above shoulder: 0.9 (0.6; 1.4) Both: 1.8 (1.2; 2.7) Women: Exposed < 2 h: 1.0 (reference) Abducted: 1.3 (0.8;1.9) Above shoulder: 0.9 (0.6; 1.5) Both: 1.2 (0.7; 2.2) Shoulder pain with RCS: Men Exposed < 2 h: 1.0 (reference) Abducted: 1.1 (0.6; 2.1) Above shoulder: 2.4 (1.4; 4.1) Both: 2.6 (1.2; 2.7) Women: Exposed < 2 h: 1.0 (reference) Abducted: 1.8 (1.0; 3.4) Above shoulder: 1.2 (0.6; 2.4) Both: 3.1 (1.5; 6.7)
Bodin (2012b) PC *Q* = 51 clinical assessment	Incidence of rotator cuff syndrome during follow-up	Working with arms abducted > 60° > 2 h/day Work with arms above the shoulder > 2 h/day	Incidence (%) arms abducted > 2 h/day: Men: no: 6.2, yes: 5.9 (*p* = 0.844) Women: no: 5.5, yes: 12.7 (*p* = 0.003)	OR for being an incident case arms abducted > 2 h/day: Men: not significant—results not reported Women: no 1.0 (reference) Yes 2.6 (1.4; 5.0) If analysis restricted to subjects without shoulder pain at baseline Women: no 1.0 (reference) Yes 3.3 (1.6; 6.9)
			Arms above shoulder: Men: no: 5.3, yes: 12.8 (*p* = 0.004) Women: no: 6.9, yes:11.3 (*p* = 0.202)	Arms above shoulder: Men: no 1.0 (reference) Yes 2.2 (1.0–4.7) Women: not significant—results not reported
Bodin (2012c) PC *Q* = 53	Incidence of shoulder pain (pain free at baseline, pain past 7 days at follow-up)	Working with arms abducted > 60° > 2 h/day Work with arms above the shoulder (yes/no)	Incidence (%) Arms abducted > 2 h/day: Men: no: 11.2, yes: 10.7 (*p* = 0.884) Women: no: 20.2, yes: 23.2 (*p* = 0.530) Arms above shoulder: Men: no: 9.5, yes: 13.8 (*p* = 0.041) Women: no: 18.7, yes: 24.7 (*p* = 0.073)	OR for being an incident case Arms abducted > 2 h/day: Men and women: Not significant—results not reported Arms above shoulder: Men: 1.5 (1.0;2.3) Women: not significant—results not reported
Bovenzi (2015) PC *Q* = 72	Shoulder pain in the past 12 months (i) episodes (ii) duration (iii) intensity	Work with hands and arms raised above shoulder level during a typical working day: “never” “< 1 h/day” “> 1 h/day”	Not available	Only male participants Episodes: Never 1.00 (reference) < 1 h/day 0.93 (0.18; 4.88) > 1 h/day 2.00 (1.02; 3.92) Duration: Never 1.00 (reference) < 1 h/day 1.89 (0.99; 3.58) > 1 h/day 1.29 (0.27; 6.18) Intensity: Never 1.00 (reference) < 1 h/day 0.97 (0.18; 5.16) > 1 h/day 2.38 (1.19; 4.78)
Descatha (2012) PC *Q* = 46	Moderate or severe shoulder pain in the past 12 months At follow-up analyzed for moderate pain among workers with no pain at baseline, and severe pain among workers with no or moderate pain at baseline Analyses at baseline (cross-sectional) also presented	Duration arm elevation > 90° without carrying loads < 1 year; 1–25 years; > 25 years This study also presents results for arm elevation > 90° while carrying loads, but these results are not included here	Not available	Only male participants Moderate vs no shoulder pain: < 1 year: 1.00 (reference) 1–25 years: 1.27 (0.78; 2.07) > 25 years: 0.82 (0.30; 2.21) Severe vs no shoulder pain: < 1 year: 1.00 (reference) 1–25 years: 1.50 (0.87; 2.56) > 25 years: 0.59 (0.19; 1.83) Severe vs no or moderate pain: < 1 years: 1.00 (reference) 1–25 years: 0.67 (0.33; 1.38) > 25 years: 1.32 (0.45; 3.94) Analyses at baseline (CS) Moderate vs no shoulder pain: < 1 years: 1.00 (reference) 1–25 years: 1.27 (0.75; 2.13) > 25 years: 1.27 (0.55; 2.93) Severe vs no shoulder pain: < 1 years: 1.00 (reference) 1–25 years: 1.45 (0.98;2.17) > 25 years: 0.75 (0.36; 1.58) Severe vs moderate or no pain: < 1 years: 1.00 (reference) 1–25 years: 1.42 (0.96; 2.10) > 25 years: 0.73 (0.35; 1.52)
Engholm (2005) CS *Q* = 47	Shoulder pain in the past 12 months Non cases: “Never” “rarely” Cases: “Often” “Very often”	How often do you work with your hands above your shoulders? “Rarely” “Rather rarely” “Sometimes” “Rather often” “Often”	Not available	Only male participants Rarely: 1.00 (reference) Rather rarely: 1.16 (1.04–1.29) Sometimes: 1.21 (1.10–1.34) Rather often: 1.64 (1.48–1.82) Often: 3.66 (3.32–4.04)
Harkness (2003) PC *Q* = 65	Pain in the shoulder complex lasting for one day or longer in the past month	Working with hands at or above shoulder level during the past working day: “Never” “< 15 min” “≥ 15 min”	Unadjusted: Never: 1.0 (reference) < 15 min 1.1 (0.7; 1.6) > 15 min 1.6 (1.2; 2.8) Univariate: Never 1.0 (reference) < 15 min 1.0 (0.7; 1.7) > 15 min 1.9 (1.2; 2.9)	Multivariate: Never: 1.0 (reference) < 15 min: 1.0 (0.6; 1.6) > 15 min: 1.6 (0.98; 2.5)
Hoe (2012) CS *Q* = 47	Shoulder pain alone in the past month Subjects with shoulder pain and neck pain as a separate category	Working with hands above shoulder > 1 h (yes/no)	Not available	Subjects with shoulder pain only: No: 1.00 (reference) Yes: 1.28 (0.67–2.46) Subjects with both shoulder pain and neck pain: No: 1.00 (reference) Yes: 1.42 (0.81–2.50)
Hooftman (2009) PC *Q* = 67	Shoulder pain or discomfort in the past 12 months	Working with hands above shoulder level: “No”, “Yes”	Not available	Men: No 1.00 (reference) Yes 1.30 (1.12; 1.52) Women: No 1.00 (reference) Yes 1.24 (1.05; 1.47)
Hoozemans (2002) CS *Q* = 53	Past year: (i) Ache, pain or discomfort in the shoulder; (ii) high shoulder pain intensity	Working with hands above shoulder heights: “Seldom or never” “Sometimes” “Quite often” “Very often”	Not available	Ache/pain/discomfort: Low exposed: 1.00 (reference) Medium exposed: 1.82 (1.13; 3.03) High exposed. n.s. (results not shown) High pain intensity: Low exposed: 1.00 (reference) Medium exposed: 2.35 (1.13; 4.86) High exposed. n.s. (results not shown)
Leclerc (2004) PC *Q* = 44	Shoulder pain for at least 1 day during the past 6 months	Working with arms above shoulder level: “Frequently” “Infrequently”	Not available	Women: Infrequently 1.00 (reference) Frequently 1.84 (0.89;3.79) Men: Factor less than *p* < 0.20 in bivariate analysis and not included in multivariate model
Luime (2004b) PC *Q* = 47	Shoulder pain or discomfort in the past 12 months	Working with hands above shoulder level: At risk: “often” or “very often” Reference: “seldom or never” or “sometimes”	Incident cases Reference: 1.00 At risk: 1.22 (0.60; 2.49), Recurrent cases Reference: 1.00 At risk: 1.13 (0.75; 1.71)	The variable “working with hands above shoulder level” did not remain in the final multivariate models
Melchior (2006) CS *Q* = 53 clinical assessment	Rotator cuff syndrome	(i) Holding one or both arms above the shoulders (ii) Holding one or both arms away from the body "Never" " < 2hours/day" " > 2hours/day"	Not available	Prevalence ratios: Arms above shoulders Men: Never: 1.00 (reference) < 2 h/day: 1.06 (0.67;1.67) > 2 h/day: 2.57 (1.67;3.97) Women: Never: 1.00 (reference) < 2 h/day: 1.21 (0.75;1.93) > 2 h/day: 1.75 (1.09;2.83) Arms away from body Men: Never: 1.00 (reference) < 2 h/day: 1.49 (0.96;2.30) > 2 h/day: 1.42 (0.87;2.31) Women: Never: 1.00 (reference) < 2 h/day: 1.23 (0.69;2.09) > 2 h/day: 2.13 (1.36;3.33)
Miranda (2005) CS *Q* = 78 Clinical assessment	Chronic rotator cuff tendinitis	Duration (years) of working with a hand above shoulder level (> 1 h/day)	Not available	Total None 1.0 (reference) 1–3 years: 2.4 (1.0;5.9) 4–13 years: 3.2 (1.6;6.5) 14–23 years: 4.7 (2.4;9.1) > 23 years: 2.3 (1.1;4.9) Men None 1.0 (reference) 1–3 years: 3.1 (1.1;8.4) 4–13 years: 3.0 (1.2;7.7) 14–23 years: 4.8 (1.9;12.1) > 23 years: 2.3 (0.7;7.0) Women None 1.0 (reference) 1–3 years: 1.0 (0.2;4.6) 4–13 years: 2.2 (0.6;7.4) 14–23 years: 4.4 (1.5;12.4) > 23 years: 2.5 (0.8;7.9)
Miranda (2001) PC *Q* = 44	Days with shoulder pain in the past 12 months Incident case: < 8 days at baseline and ≥ 8 days at follow-up Persistent case: > 30 days both at baseline and follow-up	Work with a hand above shoulder level: < 0.5 h/day 0.5–1 h/day > 1 h/day	Incidence: < 0.5 h/day 1.0 (reference) 0.5–1 h/day: 1.4 (1.0;2.0) > 1 h/day: 1.8 (1.3;2.6) Persistence: < 0.5 h/day 1.0 (reference) 0.5–1 h/day: 1.4 (0.8;2.4) > 1 h/day: 1.5 (0.8;2.5)	Incidence: < 0.5 h/day 1.0 (reference) 0.5–1 h/day: 1.1 (0.8;1.6) > 1 h/day: 1.3 (0.8;1.9) Persistence: < 0.5 h/day 1.0 (reference) 0.5-1 h/day: 1.4 (0.8;2.4) > 1 h/day: 1.4 (0.8;2.5)
Nahit (2001) CS *Q* = 44	Shoulder pain experienced during the past month, lasting > 24 h	Working with hands above shoulder level during the last working day: > 15 min (yes/no)	(Adjusted for age and sex) No: 1.0 (Reference) Yes: 1.3 (0.9;1.8)	
Niedhammer (1998) CS *Q* = 44	Shoulder disorders in the past 6 months (pain, stiffness, or discomfort), pain duration	Working with arms above shoulder level: “Frequently” “Infrequently”	Not available	Only female participants Shoulder disorders: no associations with *p* < 0.15 found Chronic shoulder disorders: Either side Infrequently 1.00 (reference) Frequently 1.94 (0.83;4.54) Left side Infrequently 1.00 (reference) Frequently 6.51 (2.07;20.05) Right side no associations with *p* < 0.15 found
Roquelaure (2011) CS *Q* = 58 clinical assessment	Rotator cuff syndrome	Working with arms abducted > 60° and/or above shoulder level ≥ 2 h/day	Men: Exposed < 2 h: 1.0 (ref) Abducted: 1.5 (0.8–2.7) Above should: 3.2 (2.0–5.2) Both: 3.1 (1.8–5.5) Women: Exposed < 2 h: 1.0 (ref) Abducted: 2.4 (1.4–4.2) Above should: 1.7 (0.9–3.3) Both: 3.9 (2.0–7.7)	Men: Exposed < 2 h: 1.0 (reference) Abducted: 0.9 (0.5;1.8) Above shoulder: 2.3 (1.3;3.9) Both: 2.0 (1.1–3;7) Women: Exposed < 2 h: 1.0 (reference) Abducted: 1.8 (1.0;3.2) Above shoulder: 1.6 (0.8;3.1) Both: 3.6 (1.8;7.3)
Seidler (2011) CC *Q* = 57 clinical assessment	Partial or total supraspinatus tendon tears	Cumulative lifetime exposure to work above shoulder level	Only male participants Never 1.0 (reference) > 0 to < 610 h 1.7 (1.0;2.8) 610 to < 3195 h 2.6 (1.6;4.2) 3195–64,057 h 4.1 (2.6;6.4)	Only male participants Never 1.0 (reference) > 0 to < 610 h 1.0 (0.6;1.8) 610 to < 3195 h 1.4 (0.8;2.4) 3195–64,057 h 2.0 (1.1;3.5)
Sim (2006) CS *Q* = 44	Shoulder pain in the last 4 weeks lasting 1 day or longer	Working with one/both arms at shoulder height or above on most or all days of the working week (yes/no)	Not available	No: 1.0 (reference) Yes: 1.1 (0.9;1.4)

Study design: *PC* prospective cohort, *CC* case–control, *CS* cross-sectional

*Q* quality score

**Table 5:** Results from studies using expert ratings to assess work with elevated arms: alphabetic order

	Outcome	Exposure	OR (95% CI)	
			Univariate	Multivariate
Dalbøge (2018a) PC *Q* = 63 clinical assessment	Surgery for subacromial impingement syndrome	Years of arm elevation > 90° for > 2 min/day	not available	All subjects: 2.0 (1.7;2.3) Analyses restricted to subjects with more than 5, 7 or 10 years job exposure: > 5 years: 1.9 (1.6;2.2) > 7 years: 1.9 (1.7;2.1) > 10 years: 2.1 (1.8;2.4)
Dalbøge (2017) CC *Q* = 76 clinical assessment	Surgery for subacromial impingement syndrome	Arm elevation years	Men 0 years: 1.0 (reference) > 0–10 years: 2.0 (1.6;2.5) > 10–60 years: 2.3 (1.8;3.0) Women 0 years: 1.0 (reference) > 0–10 years: 1.6 (1.3;1.9) > 10–60 years: 1.9 (1.4;2.6)	Men 0 years: 1.0 (reference) > 0–10 years: 2.0 (1.5;2.5) > 10–60 years: 2.3 (1.8;3.0) Women 0 years: 1.0 (reference) > 0–10 years: 1.5 (1.2;1.9) > 10–60 years: 1.9 (1.4;2.6)
Dalbøge (2014) PC *Q* = 58 clinical assessment	Surgery for subacromial impingement syndrome	Duration > 90° = > 1 arm-elevation year = work with arms > 90° for 0.5 h/day for 1 year	0 years: 1.0 (reference) > 0–2 years: 1.3 (1.3–1.4) > 2–5 years: 1.4 (1.3–1.5) > 5–10 years 1.7 (1.6–1.8) > 10–56 years 1.9 (1.8–2.0)	0 years: 1.0 (reference) > 0–2 years: 1.4 (1.4;1.5) > 2–5 years: 1.5 (1.5;1.6) > 5–10 years: 1.8 (1.7;1.9) > 10–56 years: 2.1 (2.0;2.2)
Svendsen (2013) PC *Q* = 53 clinical assessment	Surgery for subacromial impingement syndrome	Working hours/day with arm elevation > 90° at baseline (also analyzed for combination of arm elevation and neck-shoulder pain (NSP) at baseline)	Hazard ratio: 0 h/day: 1.00 (reference) > 0-1 h/day 1.60 ≥ 1 h/day 1.98 Hazard ratio: NSP-/0 h/day 1.00 (reference) NSP-./ > 0-1 h/day: 1.43 NSP-/ > 1 h/day: 2.66 NSP + ./0 h/day: 2.61 NSP + ./ > 0-1 h/day: 4.68 NSP + / > 1 h/day: 4.25	Hazard ratio: 0 h/day: 1.00 (reference) > 0-1 h/day 1.53 (1.14;2.05) ≥ 1 h/day 1.61 (1.06;2.45) Hazard ratio: NSP-/0 h/day 1.00 (reference) NSP-/ > 0-1 h/day: 1.41 (0.90;2.20) NSP-/ > 1 h/day: 2.15 (1.23;3.74) NSP + ./0 h/day: 2.74 (2.00;3.79) NSP + ./ > 0-1 h/day: 4.43 (3.01;6.52) NSP + / > 1 h/day: 3.38 (1.99;5.74)

Study design: *PC* prospective cohort, *CC* case–control, *CS* cross-sectional

*Q* quality score

**Table 6:** Results from observation studies using video recordings to assess work with elevated arms: alphabetic order

	Outcome	Exposure	OR (95% CI)	
			Univariate	Multivariate
Coenen (2016) PC *Q* = 63	Shoulder pain or discomfort in the past 12 months Analyses at baseline (cross-sectional) also presented	Total duration and maximal continuous duration of elevation ≥ 30° (hours/day)	Total duration: Not significant. Estimates not given Maximal duration: < 30° 1.00 (reference) ≥ 30° 0.85 (0.53–1.36) Analyses at baseline Total duration: Not significant. Estimates not given Maximal duration: < 30° 1.00 (reference) ≥ 30° 0.37 (0.22–0.62)	Total duration: Not significant. Estimates not given Maximal duration: < 30° 1.00 (reference) ≥ 30° 0.95 (0.57;1.59) Analyses at baseline (CS) Total duration: Not significant. Estimates not given Maximal duration: < 30° 1.00 (reference) ≥ 30° 0.50 (0.29–0.87)
Punnett (2000) CC *Q* = 73 clinical assessment	Shoulder disorder cases (total *N* = 79) or clinical cases only (at least one clinical shoulder sign, *N* = 42)	Duration of arm elevation (flexion or abduction in % of work cycle time): neutral (< 45°), mild (46–90°), severe (> 90°)	Not available	Only male participants Neutral or mild elevation: n.s Severe arm elevation (all signific.): Total cases: Left shoulder: No time OR = 1 > 0 to < 10% OR = 2.5 ≥ 10% OR = 5.1 Right shoulder: No time OR = 1 > 0 to < 10% OR = 1.7 ≥ 10% OR = 2.8 Only clinical cases: Left shoulder: No time OR = 1 > 0 to < 10% OR = 2.5 ≥ 10% OR = 6.1 Right shoulder: No time OR = 1 > 0 to < 10% OR = 2.0 ≥ 10% OR = 3.9 Exposure–response relationship: OR 1.4 (1.1–1.8) for each increment of 10% of the work cycle with severe flexion/abduction
Silverstein (2009) CS *Q* = 62 clinical assessment	Rotator cuff syndrome Shoulder symptoms: pain last seven days and occurring > week last year	Percent work time with upper arm flexion > 45°	(adjusting for age and BMI) Shoulder symptoms Men < 18% time: 1.00 (ref) > 18% time: 0.98 (0.56;1.72) Women < 18% time: 1.00 (ref) > 18% time: 1.28 (0.77;2.12) Rotator cuff syndrome Men < 18% time: 1.00 (ref) > 18% time: 1.63 (0.76;3.51) Women < 18% time: 1.00 (ref) > 18% time: 3.12 (1.27;7.68)	The variable “Percent work time with upper arm flexion > 45°” did not remain in the final multivariate models An exposure combining “work time with upper arm flexion > 45°” and “use of forceful pinch” was significantly associated with rotator cuff syndrome
Silverstein (2008) CS *Q* = 64 clinical assessment	Rotator cuff syndrome	Percent work time with upper arm flexion > 45°	(adjusting for age, gender and BMI) Upper arm flexion > 45° < 18% time: 1.00 (ref) > 18% time: 2.16 (1.22;3.83)	The variable “Percent work time with upper arm flexion > 45°” did not remain in the final multivariate models An exposure combining “work time with upper arm flexion > 45°” and “forceful exertions” was significantly associated with rotator cuff syndrome
Smith (2009) PC *Q* = 67	Shoulder pain at follow-up	Duration of upper arm: (i) Extension > 5° or flexion ≥ 45° (ii) Abduction ≥ 30°	Not available	Extension > 5° or flexion ≥ 45°: < 20% of time: 1.00 (reference) 20–35% of time: 1.84 (1.08;3.13) ≥ 35% of time: 1.15 (0.61;2.17) Upper arm abduction not included in the multivariate model

Study design: *PC* prospective cohort, *CC* case–control, *CS* cross-sectional

*Q* quality score

**Table 7:** Results from studies using inclinometers to assess work with elevated arms: alphabetic order

	Outcome	Exposure	OR (95% CI)	
			Univariate	Multivariate
Dalbøge (2017, 2018a) See Table 5 (expert ratings)		Expert decided job exposure matrix (JEM) calibrated with inclinometer measurements		
Hanvold (2015) PC *Q* = 67	Shoulder pain in the past 4 weeks	Percentage of working time > 60°, > 90° Percentage of working time > 60°, > 90° lasting at least 5 s	Risk ratio: Men: > 60°: 0.98 (0.91–1.06) > 60°, > 5 s: 0.98 (0.90–1.07) > 90°: 0.97 (0.87–1.09) > 90°, > 5 s: 0.98 (0.83–1.16) Women: > 60°: 1.23 (1.13–1.34) > 60°, > 5 s: 1.71 (1.41–2.07) > 90°: 1.72 (1.20–2.45) > 90°, > 5 s: 3.50 (1.67–7.35)	Risk ratio: Men: > 60°: 1.04 (0.96–1.14) > 60°,> 5 s: 1.05 (0.95–1.15) > 90°: 1.04 (0.93–1.17) > 90°, > 5 s: 1.05 (0.89–1.22) Women: > 60°: 1.28 (1.13–1.46) > 60°, > 5 s: 1.99 (1.54–2.59) > 90°: 1.44 (1.02–2.03) > 90°, > 5 s: 3.41 (1.49–7.81)
Koch (2017) PC *Q* = 56	Shoulder pain baseline (T1) 6 months (T2)	Percentage of work day with upper arm inclination > 30° > 60° > 90° > 120°	Regression coefficients > 30° T1: − 0.02 (− 0.03; − 0.00) T2: − 0.02 (− 0.03; − 0.00) > 60° T1: − 0.02 (− 0.05; 0.00) T2: − 0.02 (− 0.05; − 0.00) > 90° T1: − 0.03 (− 0.07; 0.01) T2: − 0.04 (− 0.09;0.00) > 120° T1: − 0.45 (− 0.71; − 0.19) T2: − 0.37 (− 0.64; − 0.10)	Regression coefficients > 30° T1: − 0.02 − 0.03; − 0.00) T2: − 0.02 (− 0.03; − 0.00) > 60° T1: − 0.02 (− 0.05;0.01) T2: − 0.02 (− 0.05;0.01) > 90° T1: − 0.04 (− 0.11;0.02) T2: − 0.04 (− 0.11;0.03) > 120° T1: − 0.31 (− 0.61; − 0.01) T2: − 0.22 (− 0.53;0.09)
Nordander (2016) CS *Q* = 58 clinical assessment	Shoulder complaints during the past 12 months and the past 7 days Clinical diagnoses in the right shoulder	Distribution of angle amplitude 50th and 99th percentile during a full work day	No statistically significant association found. Actual associations not reported	No statistically significant association found. Actual associations not reported
Svendsen (2004a) CS *Q* = 75 clinical assessment	Supraspinatus tendinitis Shoulder pain with disability Shoulder pain without disability	Current exposure—% of working hours with arm > 90° Lifetime exposure—full time working months with upper arm > 90°	Only male participants Current exposure—unadjusted estimates not provided Lifetime exposure—only crude OR without 95% CI provided Lifetime exposure (months) and supraspinatus tendinitis (dominant shoulder): 0–6 months: 1.00 6–12 months: 0.80 12–24 months: 1.33 > 24 months: 2.74 Lifetime exposure (months) and shoulder pain with disability (dominant shoulder): 0–6 months: 1.00 6–12 months: 1.05 12–24 months: 1.92 > 24 months: 3.74 Lifetime exposure (months) and shoulder pain without disability (dominant shoulder): 0–6 months: 1.00 6–12 months: 1.07 12–24 months: 1.64 > 24 months: 1.45	Only male participants Current exposure and supraspinatus tendinitis: 0–3%: 1.00 (reference) 3–6%: 0.94 (0.37;2.39) 6–9%: 4.70 (2.07;10.68) Trend (increment 1%): 1.23 (1.10;1.39) Current exposure and shoulder pain with disability: 0–3%: 1.00 (reference) 3–6%: 2.10 (1.34;3.28) 6–9%: 3.47 (2.02;5.97) Trend (increment 1%): 1.16 (1.08;1.24) Current exposure and shoulder pain without disability: 0–3%: 1.00 (reference) 3–6%: 1.22 (0.91;1.65) 6–9%: 1.84 (1.30;2.59) Trend (increment 1%): 1.08 (1.04;1.13) Lifetime exposure (months) and supraspinatus tendinitis (dominant shoulder): 0–6 months: 1.00 (reference) 6–12 months: 0.73 (0.27;1.94) 12–24 months: 1.30 (0.57;2.99) > 24 months: 1.87 (0.79;4.44) Trend (increment 6 months): 1.14 (0.97;1.35) Lifetime exposure (months) and shoulder pain with disability (dominant shoulder): 0–6 months: 1.00 (reference) 6–12 months: 1.04 (0.56;1.93) 12–24 months: 1.75 (1.03;2.97) > 24 months: 2.23 (1.28;3.88) Trend (increment 6 months): 1.18 (1.06;1.30) Lifetime exposure (months) and shoulder pain without disability (dominant shoulder): 0–6 months: 1.00 (reference) 6–12 months: 1.11 (0.74;1.70) 12–24 months: 1.68 (1.16;2.44) > 24 months: 1.85 (1.16;2.94) Trend (increment 6 months): 1.16 (1.06;1.27)
Svendsen (2004b) CS *Q* = 67 clinical assessment	Supraspinatus tendinopathy (ST), Acromioclavicular joint degeneration (AC)	Lifetime working months with upper arm elevation > 90°	Only male participants ST: 0–10 months 1.00(reference) 10–20 months 0.95 > 20 months 2.38 Trend (increment 5 months): Continuous 1.29 AC: 0–10 months 1.00(reference) 10–20 months 0.80 > 20 months 0.53 Trend (increment 5 months): Continuous 0.83	Only male participants ST: 0–10 months 1.00 (reference) 10–20 months 0.95 (0.41;2.20) > 20 months 2.33 (0.93;5.84) Trend (increment 5 months): Continuous 1.27 (1.02;1.60) AC: 0–10 months 1.00 (reference) 10–20 months 0.79 (0.35;1.77) > 20 months 0.49 (0.19;1.23) Trend (increment 5 months): Continuous 0.80 (0.64;1.00)

Study design: *PC* prospective cohort, *CC* case–control, *CS* cross-sectional

*Q* quality score

The results shown in Tables 4, 5, 6 and 7 will be commented upon in the following order below each table:

1. Overview, including the direction of statistically significant associations.
2. Evaluation of size of the point estimate, significance and quality of articles based on study design, choice of exposure estimate and outcome, use of confounders, and total quality score.
3. Gender differences.
4. When possible only including point estimates from high-quality articles (score > 50%) with prospective design focusing on arm elevation > 90° and clinical diagnoses.

### Articles using questionnaires or interviews to evaluate work exposure

Of the 20 articles using questionnaires or interviews to assess self-reported work with elevated arms, 15 used outcome based on self-reports only, four used a diagnosis of rotator cuff syndrome, and one article used partial or total supraspinatus tendon tears (Table 4).

A majority of examined associations (that are published) between self-reported exposure and effect showed positive associations (Table 4), and approximately 2/3 of these were statistically significant. Some articles showed no or negative associations between exposure and effect; however, none of the negative associations was significant. Most negative associations were found for the lowest exposure levels, except for one article that found negative associations for the highest exposure, i.e., self-reported arm-elevation > 90° for more than 25 years (Descatha et al. 2012).

High point risk estimates (OR, prevalence ratio in one study) at two or above was found in 10 of the 20 articles using self-reported exposure assessment. Only two of these ten articles had prospective design, whereas this was the case for seven out of ten articles that lacked high point estimates. On the other hand, all the articles with high point estimates were statistically significant (10 of 10 vs 2 of 10 for studies with risk estimate < 2), had higher quality score (mean 57.1 vs 50.1), more often used clinical diagnoses as outcome (6 of 10 vs 1 of 10), and used a question indicating arm elevation > 90° (5 of 10 vs 3 of 10).

Totally, 13 of the 20 articles using self-reported exposure assessment reported statistically significant positive associations between exposure and shoulder disorders. No articles reported statistically significant negative associations. All six articles using clinical diagnoses as outcome found statistically significant positive associations.

Most articles present risk estimates for both genders separately, a few only for males (Bovenzi 2015; Descatha et al. 2012; Engholm and Holmström 2005), one article only for females (Niedhammer et al. 1998), and others presented analyses independent of gender (Hoozemans et al. 2002; Miranda et al. 2001; Nahit et al. 2001). Increased risk estimates were found for both genders.

Most articles used the following question to describe exposure: “work with hands above shoulder level” or similar. The answer alternatives were yes/no or different durations of exposure as > 15 min, > 1 h or > 2 h per shift. In one article, subjects were asked if they worked with shoulder abducted > 90° for > 2 h per day (Roquelaure et al. 2011), and in another article, if they have been working with arms in that position for 1–25 years or > 25 years (Descatha et al. 2012). One study showed in several articles that “work with arms above shoulder level” (which may be interpreted as arm elevation > 90°) was associated with shoulder pain and rotator cuff syndrome (Bodin et al. 2012a, b, c).

Seidler and co-workers examined the associations with partial or total supraspinatus tendon tears (Seidler et al. 2011). They found positive associations with the cumulative lifetime exposure to work above shoulder level.

### Articles using expert ratings to evaluate work exposure

All articles in this category evaluated the association of work exposure with first-time surgery for subacromial impingement syndrome (Table 5), and all articles calculated their exposure estimates from a similar job-exposure matrix based on expert ratings (Svendsen et al. 2013). For the two most recent articles (Dalbøge et al. 2017, 2018a), the exposure estimates for some of the job titles in this job-exposure matrix had been calibrated with technical measurements (inclinometers) in whole-day field recordings. Three articles estimated lifetime cumulative exposure, while the last article (Svendsen et al. 2013) used a measure of exposure intensity at baseline. Exposure and background data were calculated from a database of previous studies (Svendsen et al. 2013), the entire Danish working population (Dalbøge et al. 2014, 2018a), or a nested case–control study based on a selected sample from this population (Dalbøge et al. 2017).

The four included articles in this category (Table 5) reported only positive associations and all were statistically significant. Three articles were prospective with high quality (mean 58, range 53–63) and found positive associations (OR ≥ 2 represented in two of the three articles) between work duration with arms elevated > 90° and surgery of subacromial impingement syndrome (Dalbøge et al. 2014, 2018a; Svendsen et al. 2013). One case–control study (quality score 76) also showed increased risk for surgery, especially for males (OR ≥ 2) (Dalbøge et al. 2017). More than 2 min work per day with arms abducted > 90° increased the risk for outcome (Dalbøge et al. 2018a). The increased risk was found for both genders.

The results from articles that used expert ratings were thus very clear, with all articles reporting statistically significant positive associations between duration of arm elevation > 90° at work and first-time surgery for subacromial impingement syndrome. All articles have quality score ≥ 50%, have high-quality exposure assessment, especially the two most recent articles (Dalbøge et al. 2017, 2018a) that used technical measures of a subgroup of occupations together with the expert ratings in the calculation of the job-exposure matrix.

### Articles using video recordings to evaluate work exposure

Five articles used video recordings to observe and assess work exposure (Table 6), and all received a high-quality score (mean 65, range 62–69). Two articles had a prospective design, one case–control, and two articles used a cross-sectional design. Four articles found at least one statistical significant positive association. Three of these articles had clinical diagnoses as outcome.

The articles categorized in the group with video recordings for exposure assessment did not show the same clear picture as those using expert ratings. Three of five articles in this category found at least one significant OR ≥ 2, all using clinical diagnoses, only one of these evaluated arm elevation > 90° and none had a prospective design. The two articles with prospective design showed OR below 2 and 1, respectively. These two articles evaluated only arm elevation ≥ 30° (or flexion ≥ 45°) and did not use clinical assessment as outcome. The quality of articles in this category was good (mean 65, range 62–69). The increased risk was found for both genders. One case–control study with the highest quality score in this category and using arm elevation > 90° as exposure variable found OR ≥ 2 for clinical shoulder disorders (Punnett et al. 2000).

### Articles using inclinometers to evaluate exposure to work with elevated arms

All five articles in this category had high-quality scores (mean 64, range 56–73), and all articles included measures of arm elevation > 90° assessed by technical measures (inclinometry). Three of the articles reported statistically significant positive associations between arm elevation and shoulder disorders, one study (Nordander et al. 2016) did not report any associations, and the last article (Koch et al. 2017) reported statistically significant negative associations (Table 7).

Two articles with cross-sectional design estimated lifetime exposure to work with arm elevation > 90°. These two articles used clinical diagnoses as outcome, both found significant positive associations with disorders in the supraspinatus tendon (Svendsen et al. 2004a, b). Two articles had prospective design, one showed positive association for young women but not for men (Hanvold et al. 2015), and the other study showed a small negative association for a mixed group with different exposures (Koch et al. 2017). One cross-sectional study showed no significant associations (Nordander et al. 2016).

Only one article stratified on gender, finding a significant increased risk for shoulder pain in young women working with elevated arms > 90° (Hanvold et al. 2015) and the other articles only investigated men or did not stratify.

Three of the articles in this category found at least one significant point estimate of risk ≥ 2 (odds or risk ratio), one of these was a prospective study the two others cross-sectional. These were high-quality articles (mean score 69, range 67–73) that used arm elevation > 90° as exposure and clinical outcomes (except one study). The two articles that did not find risk ≥ 2 were both non-significant.

### Exposure–response relationship

Eighteen of the 34 included articles presented effect estimates for three or more levels of exposure to work with elevated arms (Table 8), thus enabling us to look for a possible exposure–response relationship, with an increasing exposure to arm elevation associated with an increased reporting of shoulder disorders. One article (Punnett et al. 2000) presented such results both regarding levels of exposure intensity (level of arm elevation amplitude) and regarding exposure duration. Two articles (Hanvold et al. 2015; Koch et al. 2017) presented data on exposure intensity only, while the remaining 15 articles gave results with three or more levels of the duration of exposure to arm elevation. Several articles were examining the duration on a daily level (e.g., hours per day or percentage of time), while others were focusing on the lifetime exposure duration (e.g., months or years).

**Table 8:** Overview of studies presenting results on exposure–effect relationship: alphabetic order

	Study design	Qual score	Intensity or duration (I or D)	Positive association found?	Positive exposure–response?	Clinical diagnosis	Results in Tables
Bovenzi (2015)	PC	72	D	Yes	Yes*	No	Table 4
Dalbøge (2018a)	PC	63	D	Yes	Yes	Yes	Table 5
Dalbøge (2017)	CC	76	D	Yes	Yes	Yes	Table 5
Dalbøge (2014)	PC	58	D	Yes	Yes	Yes	Table 5
Descatha (2012)	PC	46	D	No	No	No	Table 4
Engholm (2005)	CS	47	D	Yes	Yes	No	Table 4
Hanvold (2015)	PC	67	I	Yes	Yes	No	Table 7
Harkness (2003)	PC	65	D	Yes	Yes*	No	Table 4
Koch (2017)	PC	56	I	No	No	No	Table 7
Melchior (2006)	CS	53	D	Yes	Yes	Yes	Table 4
Miranda (2005)	CS	78	D	Yes	Yes	Yes	Table 4
Miranda (2001)	CS	44	D	Yes	Yes**	No	Table 4
Punnett (2000)	CC	73	I/D	Yes	Yes	Yes	Table 6
Seidler (2011)	CC	57	D	Yes	Yes	Yes	Table 4
Smith (2009)	PC	67	D	Yes	No	No	Table 6
Svendsen (2013)	PC	53	D	Yes	Yes	Yes	Table 5
Svendsen (2004a)	CS	75	D	Yes	Yes	Yes	Table 7
Svendsen (2004b)	CS	67	D	Yes	Yes	Yes	Table 7

Study design: *PC* prospective cohort, *CC* case–control, *CS* cross-sectional

*Yes = increased risk only shown for the highest level of exposure

**Yes = only significant in univariate analysis

All articles but two (Descatha et al. 2012; Koch et al. 2017) showed at least one statistically significant positive association between exposure and effect. Among these 16 articles, 13 articles presented results where an increasing exposure was associated with an increasing effect (shoulder disorders), indicating a possible exposure–response relationship (‘Yes’ in Table 8). Of the remaining three articles, two studies (Bovenzi 2015; Harkness et al. 2003) based on self-report also showed an increasing effect in the three steps from low to high exposure, but with this increase only seen in the last step, resembling a threshold relationship (indicated with a ‘Yes*’ in Table 8). This ‘threshold’ effect was seen in two of three measures of shoulder pain with the exposure to hands and arms raised above shoulder level reported as ‘never’, ‘< 1 h/day’, or ‘> 1 h/day’ (Bovenzi 2015), and in shoulder pain related to hands at or above shoulder level ‘never’, ‘< 15 min/day’, or ‘≥ 15 min/day’(Harkness et al. 2003). All ten articles with a clinical diagnosis as outcome reported results indicating an exposure–response relationship. Summary of the relevant effect estimates from the 18 articles can be found in Tables 4, 5, 6 and 7.

In three different articles, Dalbøge and co-workers examined the relationship between work exposure and shoulder surgery in a register-based cohort study of the entire Danish population (Dalbøge et al. 2014, 2018a) or in a nested case–control study of a selected sample from this population (Dalbøge et al. 2017). Years of exposure to arm elevation > 90° were estimated with a job-exposure matrix, yielding estimates indicating an increased risk with increasing exposure. Svendsen and co-workers (Svendsen et al. 2013) used a similar approach in a cohort from a database of previous studies, finding that an increase in hours/day of arm elevation > 90° at baseline corresponded to an increased risk for having shoulder surgery at a later point in time.

In two articles with cross-sectional design by Svendsen and co-workers, lifetime exposure (Svendsen et al. 2004b) or both lifetime and current exposure (Svendsen et al. 2004a) to work with arm elevation > 90° was analyzed with regard to shoulder abnormalities on MRI (Svendsen et al. 2004b) or clinically diagnosed supraspinatus tendinitis (Svendsen et al. 2004a). Both articles found indications for an exposure–response relationship.

Punnett and co-workers (Punnett et al. 2000) divided flexion/abduction into neutral (< 45°), mild (45–90°), or severe (≥ 90°) arm elevation, and found an exposure–response relationship. For each increment of 10% of the total work cycle with severe flexion/abduction, the OR for shoulder disorder increased by 1.4.

Smith and co-workers (Smith et al. 2009) followed workers for a year and had their main focus on psychosocial factors and shoulder symptom development, while controlling for physical factors. In a multivariate model, the hazard ratio for working between 20 and 35% of work time with upper arm flexed ≥ 45° (or extended > 5°) risk for reporting shoulder pain was significantly increased, compared to working less than 20% of time with this exposure. Working more than 35% was also increased compared to less than 20%, but the estimated effect was less and not significant. Thus, this study did not show an exposure–response relationship.

In a case–control study, Seidler and co-workers (Seidler et al. 2011) recruited male patients with radiographically confirmed lesions of the supraspinatus tendon, and based on self-report cumulative, lifetime exposure was estimated for both cases and controls. They found an exposure–response relationship with the exposure categories ‘No work above shoulder level’, ‘> 0 to < 610 h’, ‘610 to < 3195 h’, ‘3195–64057 h’.

In an article with cross-sectional design, Miranda and co-workers (Miranda et al. 2005) studied determinants for clinically diagnosed chronic rotator cuff tendinitis. Self-reported number of years (none, 1–3, 4–13, 14–23, > 23) working with hand above shoulder height showed an exposure–response relationship, apart from the > 23 years of exposure category giving somewhat lower estimates than the 14–23 year category.

Hanvold and co-workers (Hanvold et al. 2015) used inclinometers and examined risk ratios for shoulder pain in arm elevation > 60° and > 90° with reference to < 60° and showed a positive exposure–response relationship for women but not for men. The risk increased even more if only work elevation periods with at least 5 s duration were included.

Koch and co-workers (Koch et al. 2017) also used inclinometers and examined associations between work duration at > 30°, > 60°, > 90°, and > 120° and shoulder pain, showing mostly small and negative associations. The results indicated an opposite trend of the exposure–response relationship compared to the Hanvold study; especially arm elevation > 120° showed a higher negative association.

## Discussion

All included articles are summarized in Table 9. They are divided between articles reporting at least one point risk estimate ≥ 2 and those reporting only lower risk estimates. The articles are grouped by the four exposure assessment methods method used; self-reported (questionnaire), expert-rated, use of observational methods (video), or technical measurements (inclinometry). The table shows design, quality score, if severe arm elevation (> 90°) was evaluated, if clinical outcome was used and statistical significant results were found and if an exposure–response relationship was found. Our assumption is that studies that use technical measures in the exposure estimates and clinical diagnoses as outcome have the highest potential validity (Winkel and Mathiassen 1994; Wærsted et al. 2010). The rationale for this table is to visualize the quality and the focus of the articles that found a large effect that may give a clinical relevant increased risk (OR ≥ 2) (Guyatt et al. 2011c). In this context, quality includes proper design, valid exposure estimate of an awkward posture (“severe arm elevation”), and documented exposure–response relationship.

**Table 9:** Included articles grouped according to exposure measurement method and according to having at least one effect with odds ratio (OR) ≥ 2

OR ≥ 2	Design	Qual	> 90°	Clin	Sign	Ex/res	OR < 2	Design	Qual	> 90°	Clin	Sign	Ex/res
Self-report							Self-report						
Bodin (2012a)	CS	58	X	X	+		Bodin (2012c)	P	53	X	X	+	
Bodin (2012b)	P	51	X	X	+		Descatha (2012)	P	46	X			No
Bovenzi (2015)	P	70			+	Yes	Harkness (2003)	P	51			+ *	Yes
Engholm (2005)	CS	47			+	Yes	Hoe (2012)	CS	47				
Hoozemans (2002)	CS	53			+		Hooftman (2009)	P	67			+	
Melchior (2006)	CS	53	X	X	+	Yes	Leclerc (2004)	P	44	X			
Miranda (2005)	CS	73			+	Yes	Luime (2004b)	P	47				
Niedhammer (1998)	CS	44	X	X	+		Miranda (2001)	P	44				Yes**
Roquelaure (2011)	CS	58	X	X	+		Nahit (2001)	CS	44				
Seidler (2011)	CC	51			+	Yes	Sim (2006)	CS	44	X			
Mean quality		56					Mean quality		49				
Expert rating							Expert rating						
Dalbøge (2018a)	P	63	X	X	+	Yes	Svendsen (2013)	P	53	X	X	+	Yes
Dalbøge (2017)	CC	76	X	X	+	Yes							
Dalbøge (2014)	P	58	X	X	+	Yes							
Mean quality		65					Mean quality		53				
Video recording							Video recording						
Punnett (2000)	CC	69	X	X	+	Yes	Coenen (2016)	P	63				
Silverstein (2009)	CS	62		X	+		Smith (2009)	P	67			+	No
Silverstein (2008)	CS	64		X	+								
Mean quality		65					Mean quality		65				
Inclinometry							Inclinometry						
Hanvold (2015)	P	67	X		+	Yes	Koch (2017)	P	54	X			No
Svendsen (2004a)	CS	73	X	X	+	Yes	Nordander (2016)	CS	58		X		
Svendsen (2004b)	CS	67	X	X	+	Yes							
Mean quality		69					Mean quality		56				

OR ≥ 2: At least one odds/risk/hazard/prevalence ratio above 2 for relationship with work with elevated arms. Without this: OR < 2

Design: study design: *P* prospective, *CC* case–control, *CS* cross-sectional

Qual Quality score in % of maximal obtainable score

> 90°: Give results for work with upper arms (elbows) above 90°

Clin: Outcome by clinical examination

Sign: +  = at least one statistically significant positive relationship [+ * = OR 1.6 (0.98; 2.5)]

Ex/res: Yes or no states the results concerning exposure–response relationship. Yes** = relationship only significant in univariate analysis

On one side, articles that document higher risk estimates (≥ 2) have higher quality score, include analyses of severe arm elevation, more often use clinical outcome, and report an exposure–response relationship compared to articles reporting lower risk estimates. All these articles found statistically significant positive associations. On the other side, Table 9 shows that prospective articles more frequently report lower risk estimates and some of the articles using the most valid exposure assessments found no positive associations or even small negative associations. These two aspects may reduce the strength of the evidence. However, the three articles using self-reported exposure and finding a statistically significant OR < 2 had the highest quality score in that category (Bodin et al. 2012c; Harkness et al. 2003; Hooftman et al. 2009).

Overall, the articles using “objective” exposure assessments and found large effects were also designed to evaluate risk factors for shoulder disorders specifically (except the studies by Silverstein et al.). The three articles using “objective” exposure assessment methods that found no association between arm elevation and shoulder disorders were all performed on mixed populations with a few subjects with high exposure (Coenen et al. 2016; Koch et al. 2017; Nordander et al. 2016). They were not designed to focus on shoulder disorders specifically, and may, therefore, lack the contrast of exposure data, that is necessary to detect differences.

An exposure–response relationship was found in many high-quality articles when relating exposure intensity of arm elevation (level of arm elevation, amplitude) as well as the duration of arm elevation, especially > 90°, with both shoulder pain and clinical diagnoses. However, there is no consensus of a “safe level” for arm elevation.

If only articles with prospective or case–control designs were included in the review, a majority of articles would have reported ORs below 2. However, in total, 13 of 19 articles with these designs showed a statistically significant positive association between exposure and effect.

If only articles examining arm elevation > 90° were included, 13 out of 17 showed a statistically significant positive association between exposure and effect.

If only articles using clinical outcome were included, 15 of 16 showed a statistically significant positive association between exposure and effect.

We conclude that the documentation up to date shows a limited evidence for an association between arm elevation at work and shoulder disorders. This is based on 24 out of 34 articles that found a statistically significant positive association between exposure and effect. However, several of the articles (*N* = 15) finding a smaller effect (OR < 2) were insignificant but with a prospective design. This decreases the grade of evidence from moderate to limited.

Severe arm elevation with elbows above shoulder level (i.e. > 90°) shows a moderate evidence for an association with shoulder disorders. The higher grade of evidence with arm elevation > 90° is motivated by the higher ORs (larger effects, OR ≥ 2) and more commonly documented exposure–response relationship compared to smaller effects (OR < 2). Twelve out of nineteen articles that found ORs ≥ 2 examined severe arm elevation and 12 of the 19 studies finding larger effects also found an exposure–response relationship.

The findings cover both shoulder pain and clinical diagnosed shoulder disorders. Thirteen of the 19 articles that found a large effect used clinical diagnoses as main outcome. The strength of evidence is moderate for an exposure–response relationship between both intensity/level and duration of arm elevation at work and shoulder disorders. No cut-off level for a “safe” exposure was possible to establish.

### Limitations

#### Methodological considerations concerning included documentation

The included articles were estimated to be of sufficient quality to give valid results. Fifteen of the 34 included articles have a cross-sectional design, which make it difficult or impossible to evaluate the time dimension, exposure before outcome. This concerns especially the articles with simultaneous self-reported exposure and outcome assessments, introducing the possibility for differential misclassification (Engholm and Holmström 2005; Hoe et al. 2012; Hoozemans et al. 2002; Miranda et al. 2005; Nahit et al. 2001; Sim et al. 2006). Removing these six articles did not change the distribution on categories between large or small effects, results or quality score, except that the three articles in the small-effect category had lower quality score level (45% vs 49% for the whole category) (Hoe et al. 2012; Nahit et al. 2001; Sim et al. 2006). The other cross-sectional studies used clinical diagnoses as outcomes and had self-report of exposure only, though keeping the problem with the time dimension. Most of the articles in the other exposure assessment categories (expert rating, video, and inclinometry) have a case–control or a prospective design. These include point estimates of arm elevation exposure performed by video (Coenen et al. 2016; Punnett et al. 2000; Smith et al. 2009). Punnett and co-workers and Smith and co-workers performed short-term video recordings on representative job cycles in occupations with cyclic pattern. Coenen and co-workers (Coenen et al. 2016) performed short-term recordings in many different occupations, also non-cyclic. This latter condition may reduce the validity and representativity of exposure assessments.

The article by Coenen and co-workers reported negative associations between maximal continuous duration of arm elevation (≥ 30°) and shoulder pain. This association was only statistically significant for a cross-sectional analysis at baseline in their prospective cohort study (Coenen et al. 2016). The reported measure ‘maximal continuous duration of arm elevation’ may be conceived as a measure of peak exposure.

One-day full-shift inclinometry measurements were performed on workers with non-cyclic tasks (Hanvold et al. 2015; Koch et al. 2017). These exposure estimates are vulnerable for day-to-day variations, but assuming stable work exposure conditions, they were evaluated as valid and representative.

Our assumption was that the articles with more “objective” exposure assessment should be weighted higher compared to articles based on self-report, all other quality indications being similar. However, the issue of representativeness of technical measurements related to actual exposure during weeks or years is also important to evaluate. Four articles performed expert ratings of exposure; two of them only used this in elaboration of a job-exposure matrix (JEM) evaluating the effect of long-term exposure (Dalbøge et al. 2014; Svendsen et al. 2013). The two others used inclinometry on representative smaller samples to adjust this exposure assessment (Dalbøge et al. 2017, 2018a). By these means, the two latter articles by Dalbøge and co-workers probably offers a more valid exposure estimate over time.

Exposure–response relationship was found between exposure and outcome with no lower level of safe exposure. Expert ratings suffer from weakness due to subjective rating of a group based on job titles, however, not by the subjects individually. The strength of these articles is that they independently of subjects assess the exposure over longer time periods, not only at the time of video or inclinometry measurements. The results in this category of exposure assessments were based on four articles, all from the same Danish research group, with surgery for impingement syndrome as outcome. Another question is how the selection to surgery occurs; is it possible that subjects with impingement syndrome are more prone for operation if they need a healthy shoulder in physically heavy overhead work? However, all articles within this category were of very high quality and showed statistically significant positive effects for clinical disorders with arm elevation > 90° at work.

Two articles with cross-sectional design from the same sample of journeymen were performed by Svendsen and co-workers using inclinometry on four consecutive full shifts, resulting in a robust objective exposure assessment of arm elevation (Svendsen et al. 2004a, b). Both current and lifetime exposures were related to clinical diagnoses. Another cross-sectional study also used inclinometry to assess exposure and clinical examination to assess outcome (Nordander et al. 2016). The exposure was point estimates, mostly based on full-shift measurements. The two articles with cross-sectional design by Silverstein and co-workers were based on data from the same sample performing cyclic work and exposure was assessed by short-term video (Silverstein et al. 2008, 2009) and outcome by clinical examination. These latter three articles have only one-shift point estimates of exposure making them more vulnerable to day-to day variations.

#### Methodological considerations concerning present review

The basic question about causality will not be discussed here, but only briefly touched. This is a difficult, if not impossible, question to definitely answer. We use here the epidemiological evidence taking into account its quality and validity in making conclusions. The level of evidence for a possible causal relationship is based on a GRADE evaluation and pathophysiological studies are used to substantiate and discuss our conclusions.

Studies with a prospective design and case–control studies are weighted higher than cross-sectional studies with the aim to substantiate causal relationships. The drawback of many of the prospective studies is that they only use self-reported exposure data, where the assessment of duration is shown to be less valid (Koch et al. 2016). Cross-sectional studies of high quality are also included. The quality of the studies is taken into consideration when assessing the documentation, according to the GRADE guidelines.

Our assessment of study quality is performed using checklists that have been used at our institute for many years (Knardahl et al. 2008, 2017 ; Veiersted et al. 2017). It is based on earlier guidelines (Ariens et al. 2000; van Tulder et al. 2003). The number achieved at the quality score should be taken with some precaution. It is not a very precise estimate, and other reviews have, therefore, e.g., used three levels of quality; insufficient, moderate, or high (SBU 2014). However, we have kept the original score, without stressing the exact number in the Discussion. The appropriateness of using a general detailed scoring scheme for different types of studies has been questioned, recommending the use of a simple and specific checklist (Sanderson et al. 2007). One might argue that some of the items in our checklist are less relevant for the present review of work above shoulder level and shoulder disorders. However, using the same general scheme for quality assessment covering the most important domains in several reviews, has its merits, as long as the resulting scores are regarded as an indication and not as a final judgment of the quality level.

In our evaluation of documentations, more weight is also given to articles with well-defined outcome variables, such as clinical diagnoses.

The rather narrow focus of this review, arm elevation at work as a possible cause of shoulder disorders, sets extra demands on articles to adjust for other risk factors. Whenever possible, we use the most adjusted models for analyses of associations to be as sure as possible to extract knowledge, especially on arm elevation as a risk factor. However, when working with arms above shoulder height, the work may often include risk factors such as repetitive movements, forceful exertions, and carrying weights or heavy tools, making it difficult to establish to what extent it is the elevated arm per se, that is the main risk factor.

Two larger studies are represented by several articles. This concerns a French population study in the Loire Valley region published by Melchior and co-workers in 2006 (Melchior et al. 2006) and in several succeeding articles (Bodin et al. 2012a, b, c; Roquelaure et al. 2011). The articles used mostly the same exposure assessment (self-reported duration of work with arms above shoulder level), but had different outcomes or analytical approach. A cohort study following surgery of subacromial impingement syndrome in the Danish work force is represented by three articles (Dalbøge et al. 2014, 2017, 2018a). The outcome was identical in the Danish articles, but the exposure assessment was different. Due to the different approaches in the individual articles based on the French and Danish cohorts, we chose to keep them as individual suppliers of information.

The authors decided not to make pool data for (meta-)analyses, and instead go into detail with the different exposure assessment approaches used in the separate articles. The reason for this decision was the heterogeneity in exposure and outcome measures. However, it is also a weakness, because the variation in data and strength of associations are not shown by this approach, as it is by forest plots (Guyatt et al. 2011b).

We chose to summarize included articles in Table 9, by dividing them by assumed increased validity of exposure assessment method, but also if they showed a large effect (OR ≥ 2) of arm elevation on shoulder disorders. Evidently, by sorting the articles this way, more significant results may be expected in the large effect category. It is possible that articles showing large effects are prone to publication bias. We also found more articles on severe arm elevation in the large-effect category. A reason may be that the use of severe arm elevation promotes contrast of exposure.

By focusing on work above shoulder height, this review specifies a special kind of exposure, but still leaves some room for interpretations. On one hand, a posture with upper arm vertical hanging and maximally flexed elbow may allow the hand to work above shoulder height. On the other hand, work with arm above shoulder height, especially with the whole arm above shoulder height, constitutes probably a higher load on the shoulder structures. This means that arm elevation (assessed as the angle between the upper arm vector and the vertical line pointing downwards) is an important part of “work above shoulder height”. ‘Arms above shoulder height’ may be interpreted as the whole arm at that level (i.e., > 90°). The same regards ‘elbow above shoulder height’.

### General interpretation

#### Summary of selected previous reviews

Several reviews conclude that exposure to arm elevation at work constitutes an important risk factor for shoulder pain (Bernard 1997; Walker-Bone et al. 2003), specific shoulder disorders (Jones et al. 2007; van der Molen et al. 2017), and also when only using documentation from studies with prospective design (Mayer et al. 2012; van Rijn et al. 2010). To our knowledge, no previous review has focused exclusively on arm elevation as a possible risk factor. However, several reports and papers have included arm elevation as one of several mechanical exposures. The selection of previous reviews cited below is not based on a critical systematic search, but dependent on the authors' knowledge of the field.

A NIOSH report from 1997 (Bernard 1997) concludes with evidence for a relationship between repeated or sustained shoulder postures with more than 60 degrees of flexion or abduction and shoulder disorders. This conclusion was not found in the report from the National Research Council in 2001 (NRC 2001). Van der Windt and co-workers (van der Windt et al. 2000) concluded with inconsistent findings for awkward postures. The same year, Keyserling (Keyserling 2000) concluded that work with elevated arms constituted a significant biomechanical and psychophysical strain for the shoulder. Walker-Bone and co-workers (Walker-Bone and Cooper 2005; Walker-Bone et al. 2003) cite the Bernard report and other studies and stresses overhead work with tools as an important risk factor. Larsson and co-workers (Larsson et al. 2007) cite other reviews, stating that work with arms lifted above shoulder level was a well-documented risk factor for neck–shoulder disorder. Van Rijn and co-workers (van Rijn et al. 2010) concluded in a review with an association between “hands above shoulder” and clinical diagnoses of the shoulder. Mayer and co-workers (Mayer et al. 2012) reviewed longitudinal studies in an attempt to substantiate a causal relationship and found ORs between 1.1 and 1.8 (mostly non-significant) for work with “hands above shoulder level” and shoulder complaints. A report from the Swedish Council on Health Technology Assessment concluded in 2012 that scientific documentation provided insufficient evidence for an association between work with hands above shoulder height and shoulder disorders (SBU 2012). The National Board of Industrial Injuries and the Occupational Diseases Committee in Denmark (Arbejdsskadestyrelsen) published in 2007 a review of associations between work-related exposure and rotator cuff disease and/or biceps tendinitis (Jones et al. 2007). The authors concluded that there is moderate-to-strong evidence to suggest a causal relationship between working with arms in an elevated position and rotator cuff disease/biceps tendinitis. A review with similar background that included studies up to October 2016 that focused on subacromial impingement syndrome (SIS) concluded with a moderate evidence of a causal association between arm posture and SIS (Dalbøge et al. 2018b). Van der Molen and co-workers (van der Molen et al. 2017) reviewed studies examining work-related risk factors for clinically assessed soft-tissue shoulder disorders and found moderate evidence for an association with arm–hand elevation at work. A Norwegian general review of documentation for work-related musculoskeletal disorders (Veiersted et al. 2017) concluded with high evidence in observational studies for an association between work with elevated arms (especially with elbow above shoulder level) and shoulder disorders/pain.

The overall impression from previous reviews is that work with elevated arms is associated with increased risk of shoulder disorders. However, different aspects of this exposure have been mixed and pooled in different reviews. The actual shoulder load of “elevated arm in general” or “working with hands above shoulder” is very different from “work with elbow above shoulder level”, i.e., abducted arm > 90°. The present review attempts to take the shoulder load into consideration when evaluating the documentation for a possible causal relationship.

#### Other risk factors

Studies addressing the possible relationship between work with elevated arms and shoulder pain and disorders need to control for other risk factors associated with shoulder disorders to reduce bias. An increase of shoulder complaints with increasing age is well documented (Bernard 1997; Bodin et al. 2012c) as well as a higher prevalence in females (Hooftman et al. 2009). Other individual factors may also be associated with shoulder disorders, such as, e.g., leisure-time sport activities (Bernard 1997), smoking, obesity, and metabolic syndrome (Rechardt et al. 2010).

Several mechanical workload factors may moderate or increase the shoulder load and thereby the risk for shoulder disorders. This concerns arm elevation combined with force use, e.g., handling of tools or heavy loads (Andersen et al. 2007; Bodin et al. 2012b; Descatha et al. 2012), work with handheld vibrating tools (Seidler et al. 2011) and repetitive movements of the shoulder (Andersen et al. 2003; Herin et al. 2014). Highly repetitive work (≥ 15 movements per min) has been associated with subacromial impingement syndrome (Svendsen et al. 2013).

Psychosocial factors in the workplace have been associated with shoulder disorders, most consistently with regard to unspecific shoulder pain (Bodin et al. 2012b; van der Windt et al. 2000). More contradictory results have been reported for the association with specific disorders such as subacromial impingement syndrome (Dalbøge et al. 2018b) and rotator cuff syndrome (Roquelaure et al. 2011), and a recent a review of risk factors for impingement syndrome concluded with good evidence for no association (Dalbøge et al. 2018b). Differential association with specific shoulder disorders and non-specific shoulder pain has also been shown for personal and mechanical risk factors (Walker-Bone et al. 2006). Job demand, job control, social support, and job satisfaction are the psychosocial factors most frequently included as potential confounders (Dalbøge et al. 2018b; van der Windt et al. 2000).

In the present review, all included studies controlled for age and gender. Both the number and category (individual, mechanical, and psychosocial) of other risk factors that were included as confounders and adjusted for in the multivariate analyses varied considerably between the articles (see Appendix 4). We regarded it as a sign of quality when a study had addressed relevant risk factors for shoulder pain and disorders, other than work with elevated arms. This view was also reflected in the quality-scoring scheme used in this review. Nearly all included articles adjusted for one or more individual risk factors in addition to age and gender. Half of the articles adjusted for psychosocial risk factors, and about the same number of articles adjusted for mechanical risk factors. Approximately a third of the articles included as confounders risk factors from all three categories (individual, mechanical, and psychosocial). High physical workload was the mechanical risk factors most often included, while a few articles included repetitive work or use of handheld vibrating tools.

#### Implications for future research

Methods for improving exposure assessment should be focused upon in the future. This concerns increasing representativeness of technical measures for valid point-estimates as well as elaboration of valid job exposure matrices. Continuous variables should be used in an attempt to find “safe-levels” of exposure, if existing. Methods should be elaborated that simplify exposure assessments and standardized effective procedures for the definition of relevant outcome, both for improving the possibilities for better epidemiological studies in the future. We need more research on possible pathophysiological mechanisms to better know how to implement interventions. Breaks from continuous overhead work to promote recovery/restitution are important to counteract harmful effects; however, ideal break patterns and ideal activities in such breaks have not been established.

## Conclusions

We conclude with a limited evidence for an association between arm elevation at work and shoulder disorders, and a moderate evidence for an association between severe arm elevation with elbows above shoulder level (i.e. > 90°) and shoulder disorders. The findings covers both shoulder pain and clinical diagnosed shoulder disorders. The strength of evidence is moderate for an exposure–response relationship between both intensity/level and duration of arm elevation at work and shoulder disorders.

## Electronic supplementary material

Below is the link to the electronic supplementary material.

Supplementary file1 (DOCX 25 kb)

Supplementary file2 (DOCX 32 kb)

Supplementary file3 (DOCX 66 kb)

Supplementary file4 (DOCX 18 kb)
