# Supplementary material for: Work above shoulder level and shoulder complaints: a systematic review
Source: Int Arch Occup Environ Health. 2020 Jun 22;93(8):925–54. doi: 10.1007/s00420-020-01551-4 (PMC7519900; doi:10.1007/s00420-020-01551-4)
Supplement: Supplementary file 1 — Supplementary file1 (DOCX 25 kb) [file 420_2020_1551_MOESM1_ESM.docx]

**Appendix 1: Search strategy**

Head librarian Benedicte Mohr at the National Institute of Occupational Health gave skilful assistance with establishing the search strategy and performing the searches in the three scientific databases.

**Medline Database**

| **Nr.** | **Included terms** | **References** |
| --- | --- | --- |
| 1 | exp Shoulder joint/ | 17080 |
| 2 | Shoulder Joint/pp [Physiopathology] | 3504 |
| 3 | exp shoulder pain/ | 4016 |
| 4 | Shoulder Pain/et, pp [Etiology, Physiopathology] | 2443 |
| 5 | exp ACROMIOCLAVICULAR JOINT/ | 1860 |
| 6 | exp Joint disease/ | 345113 |
| 7 | exp shoulder dislocation/ | 5402 |
| 8 | exp bursitis/ | 4442 |
| 9 | exp TENDINOPATHY/ | 10783 |
| 10 | exp OSTEOARTHRITIS/ | 54351 |
| 11 | exp JOINT INSTABILITY/ | 17894 |
| 12 | exp rotator cuff/ | 5467 |
| 13 | Rotator Cuff/pa [Pathology] | 1245 |
| 14 | exp SHOULDER IMPINGEMENT SYNDROME/ | 1567 |
| 15 | Shoulder Impingement Syndrome/pa [Pathology] | 174 |
| 16 | exp trigger points/ | 345 |
| 17 | exp Myofascial Pain Syndromes/ | 6207 |
| 18 | MOVEMENT/ph [Physiology] | 25286 |
| 19 | (Shoulde* adj2 (disorder* or complain* or froze*)).mp. | 2050 |
| 20 | ((Glenohumeral adj2 translatio*) or arthrosis*).mp. | 5322 |
| 21 | Arthrosis.mp. | 5150 |
| 22 | (Adhesive adj2 capsuliti*).mp. | 774 |
| 23 | Capsulitis.mp. | 919 |
| 24 | Shoulder adhesive capsulitis.mp. | 49 |
| 25 | Bicipital tendinitis.mp. | 36 |
| 26 | ((Tendinitis or tendonitis) adj2 shoulder).mp. | 90 |
| 27 | (Degenerativ* adj2 (Arthritis or arthritide*)).mp. | 1329 |
| 28 | (Rotator cuff adj2 (syndrom or tear*)).mp. | 4093 |
| 29 | Rotator cuff syndrome.tw. | 76 |
| 30 | Subacromial impingement syndrome.tw. | 340 |
| 31 | 1 or 2 or 3 or 4 or 5 or 6 or 7 or 8 or 9 or 10 or 11 or 12 or 13 or 14 or 15 or 16 or 17 or 18 or 19 or 20 or 21 or 22 or 23 or 24 or 25 or 26 or 27 or 28 or 29 or 30 | 392699 |
| 32 | Shoulde* load*.mp. | 84 |
| 33 | Arm elevation.mp. | 424 |
| 34 | (upper adj2 arm adj2 elevation).tw. | 35 |
| 35 | (Hand* adj2 abov* adj2 shoulde*).mp. | 29 |
| 36 | Arm* abov* shoulde*.mp. | 14 |
| 37 | (Work* adj2 abov* adj2 shoulde* adj2 heigh*).mp. | 5 |
| 38 | (Elbow adj2 abov* adj2 shoulde*).mp. | 1 |
| 39 | (Hand* adj2 abov* adj2 shoulde*).mp. | 29 |
| 40 | (Overhead adj2 (work* or activitie* or liftin*)).mp. | 256 |
| 41 | (Shoulde* adj2 (muscl* or load*)).mp. | 1804 |
| 42 | (Arm adj2 (Repetitiv* or elevation*)).mp. | 563 |
| 43 | (Awkward adj2 shoulde* adj2 posture*).mp. | 5 |
| 44 | (workin* adj2 posture).tw. | 289 |
| 45 | (shoulde* adj2 pain*).tw. | 6996 |
| 46 | Occupational Diseases/et [Etiology] | 18972 |
| 47 | Occupational Exposure/ae [Adverse Effects] | 18154 |
| 48 | WORK/ | 19401 |
| 49 | WORKLOAD/ | 18901 |
| 50 | (work adj3 relate*).tw. | 18080 |
| 51 | work.ti. | 80215 |
| 52 | work-related.tw. | 13419 |
| 53 | 32 or 33 or 34 or 35 or 36 or 37 or 38 or 39 or 40 or 41 or 42 or 43 or 44 or 45 or 46 or 47 or 48 or 49 or 50 or 51 or 52 | 158223 |
| 54 | 31 and 53 | 7103 |
| 55 | limit 54 to yr="1990 -Current" | 6191 |

**Embase Database**

| **Nr.** | **Included terms** | **References** |
| --- | --- | --- |
| 1 | exp shoulder/ | 29799 |
| 2 | exp shoulder pain/ | 12565 |
| 3 | acromioclavicular joint/ | 1594 |
| 4 | exp shoulder dislocation/ | 3590 |
| 5 | exp bursitis/ | 3258 |
| 6 | exp tendinitis/ | 11874 |
| 7 | exp rotator cuff/ | 6232 |
| 8 | exp shoulder impingement syndrome/ | 2373 |
| 9 | exp thorax outlet syndrome/ | 1765 |
| 10 | exp trigger point/ | 1901 |
| 11 | exp myofascial pain/ | 3856 |
| 12 | (Shoulde* adj2 (disorder* or complain* or froze*)).mp. | 2931 |
| 13 | ((glenohumeral adj2 translatio*) or arthrosis*).mp. | 3494 |
| 14 | Arthrosis.mp. | 3318 |
| 15 | (Adhesive adj2 capsuliti*).mp. | 912 |
| 16 | Capsulitis.mp. | 1087 |
| 17 | shoulder adhesive capsulitis.mp. | 64 |
| 18 | Bicipital tendinitis.mp. | 23 |
| 19 | ((tendinitis or tendonitis) adj2 shoulder).mp. | 109 |
| 20 | (Degenerativ* adj2 (Arthritis or arthritide*)).mp. | 1050 |
| 21 | (Rotator cuff adj2 (syndrom or tear*)).mp. | 4370 |
| 22 | rotator cuff syndrome.tw. | 70 |
| 23 | Subacromial impingement syndrome.tw. | 411 |
| 24 | exp arthropathy/ | 495943 |
| 25 | exp osteoarthritis/ | 91514 |
| 26 | exp JOINT INSTABILITY/ | 11275 |
| 27 | 1 or 2 or 3 or 4 or 5 or 6 or 7 or 8 or 9 or 10 or 11 or 12 or 13 or 14 or 15 or 16 or 17 or 18 or 19 or 20 or 21 or 22 or 23 or 24 or 25 or 26 | 534351 |
| 28 | Shoulde* load*.mp. | 84 |
| 29 | Arm elevation.mp. | 447 |
| 30 | (upper adj2 arm adj2 elevation).tw. | 32 |
| 31 | (Hand* adj2 abov* adj2 shoulde*).mp. | 28 |
| 32 | arm* abov* shoulde*.mp. | 19 |
| 33 | (Work* adj2 abov* adj2 shoulde* adj2 heigh*).mp. | 6 |
| 34 | (Elbow adj2 abov* adj2 shoulde*).mp. | 0 |
| 35 | (Hand* adj2 abov* adj2 shoulde*).mp. | 28 |
| 36 | (Overhead adj2 (work* or activitie* or liftin*)).mp. | 281 |
| 37 | (Shoulde* adj2 (muscl* or load*)).mp. | 1848 |
| 38 | (Arm adj2 (Repetitiv* or elevation*)).mp. | 611 |
| 39 | (Awkward adj2 shoulde* adj2 posture*).mp. | 6 |
| 40 | (workin* adj2 posture).tw. | 244 |
| 41 | (shoulde* adj2 pain*).tw. | 7919 |
| 42 | occupational disease/et [Etiology] | 4927 |
| 43 | occupational exposure/ | 54668 |
| 44 | work/ | 23606 |
| 45 | workload/ | 31218 |
| 46 | (work adj3 relate*).tw. | 19067 |
| 47 | work.ti. | 49815 |
| 48 | work-related.tw. | 14221 |
| 49 | 28 or 29 or 30 or 31 or 32 or 33 or 34 or 35 or 36 or 37 or 38 or 39 or 40 or 41 or 42 or 43 or 44 or 45 or 46 or 47 or 48 | 171746 |
| 50 | 27 and 49 | 12960 |
| 51 | limit 50 to yr="1990 -Current" | 12912 |
| 52 | limit 51 to exclude medline journals | 1465 |

**Health and Scientific Abstracts Database**

Exposure

Overhead work or lifting or activity

Work

Work-related

Shoulder / Shoulder complaints

Shoulder (load* OR disorde* OR complain* OR pain* OR joint)

Hand OR arm near/2 abov* near/2 shoulde*

Shoulde* OR arm* OR upper arm* near/2(repetitiv* or elevatio*)

[ti(Shoulder (load* OR disorde* OR complain* OR pain* OR joint)) OR ti(Hand OR arm NEAR/2 abov* NEAR/2 shoulde*) OR ti(Shoulde* OR arm OR upper arm NEAR/2 (repetitiv* OR elevatio*)) AND ti(occupational disease* OR occupational exposure OR work* OR workplace* OR workload* OR work* relate*)](https://search.proquest.com/recentsearches.recentsearchtabview.recentsearchesgridview.scrolledrecentsearchlist.checkdbssearchlink:rerunsearch/C964ADEE251E4A21PQ/None?site=agricenvironm&t:ac=RecentSearches)Limits applied

ti(Shoulder (load* OR disorde* OR complain* OR pain* OR joint)) OR ti(arm NEAR/2 abov* NEAR/2 shoulde*) OR ti(Shoulde* OR arm OR upper arm NEAR/2 (repetitiv* OR elevatio*)) AND ti(occupational disease* OR occupational exposure OR work* OR workplace* OR workload* OR work* relate*)

ti(Shoulder (load* OR disorde* OR complain* OR pain* OR joint)) OR ti(arm NEAR/2 abov* NEAR/2 shoulde*) OR ti(Shoulde* OR arm OR upper arm NEAR/2 (repetitiv* OR elevatio*)) not vibration
