# Supplementary material for: Work above shoulder level and shoulder complaints: a systematic review
Source: Int Arch Occup Environ Health. 2020 Jun 22;93(8):925–54. doi: 10.1007/s00420-020-01551-4 (PMC7519900; doi:10.1007/s00420-020-01551-4)
Supplement: Supplementary file 2 — Supplementary file2 (DOCX 32 kb) [file 420_2020_1551_MOESM2_ESM.docx]

**Appendix 2: 160 articles excluded after evaluation of full-text**

Aasa, U., et al. (2005), 'Relationships between Work-related Factors and Disorders in the Neck-shoulder and Low-back Region among Female and Male Ambulance Personnel', *Journal of Occupational Health,* 47 (6), 481-89.

Adegoke, B. O., Akodu, A. K., and Oyeyemi, A. L. (2008), 'Work-related musculoskeletal disorders among Nigerian physiotherapists', *BMC Musculoskeletal Disorders,* 9, 112.

Aghili, M. M., Asilian, H., and Poursafa, P. (2012), 'Evaluation of musculoskeletal disorders in sewing machine operators of a shoe manufacturing factory in Iran', *JPMA - Journal of the Pakistan Medical Association,* 62 (3 Suppl 2), S20-5.

Al-Mohrej, O. A., et al. (2016), 'Prevalence of musculoskeletal pain of the neck, upper extremities and lower back among dental practitioners working in Riyadh, Saudi Arabia: a cross-sectional study', *BMJ Open,* 6 (6), e011100.

Alexopoulos, E. C., Burdorf, A., and Kalokerinou, A. (2003), 'Risk factors for musculoskeletal disorders among nursing personnel in Greek hospitals', *International Archives of Occupational & Environmental Health,* 76 (4), 289-94.

Alipour, A., et al. (2008), 'Occupational neck and shoulder pain among automobile manufacturing workers in Iran', *American Journal of Industrial Medicine,* 51 (5), 372-79.

Alperovitch-Najenson, D., et al. (2010), 'Upper body quadrant pain in bus drivers', *Archives of Environmental & Occupational Health,* 65 (4), 218-23.

Andersen, J. H., Haahr, J. P., and Frost, P. (2007), 'Risk factors for more severe regional musculoskeletal symptoms: a two-year prospective study of a general working population', *Arthritis & Rheumatism,* 56 (4), 1355-64.

Andersen, J. H., et al. (2003), 'Risk factors in the onset of neck/shoulder pain in a prospective study of workers in industrial and service companies', *Occupational and Environmental Medicine,* 60 (9), 649-54.

Andersen, J. H., et al. (2002), 'Physical, psychosocial, and individual risk factors for neck/shoulder pain with pressure tenderness in the muscles among workers performing monotonous, repetitive work', *Spine,* 27 (6), 660-7.

Ando, S., et al. (2000), 'Associations of self estimated workloads with musculoskeletal symptoms among hospital nurses', *Occupational & Environmental Medicine,* 57 (3), 211-6.

Anyfantis, I. D. and Biska, A. (2017), 'Musculoskeletal Disorders Among Greek Physiotherapists: Traditional and Emerging Risk Factors', *Safety and Health at Work.*

Arvidsson, I., et al. (2012), 'Discrepancies in pain presentation caused by adverse psychosocial conditions as compared to pain due to high physical workload?', *Work,* 41 Suppl 1, 2472-5.

Attar, S. M. (2014), 'Frequency and risk factors of musculoskeletal pain in nurses at a tertiary centre in Jeddah, Saudi Arabia: a cross sectional study', *BMC Research Notes,* 7, 61.

Barghout, N. H., Al-Habashneh, R., and Al-Omiri, M. K. (2011), 'Risk factors and prevalence of musculoskeletal disorders among Jordanian dentists', *Jordan Medical Journal,* 45 (2), 195-204.

Barton, N. J., et al. (1992), 'Occupational causes of disorders in the upper limb', *BMJ,* 304 (6822), 309-11.

Beach, J., Senthilselvan, A., and Cherry, N. (2012), 'Factors affecting work-related shoulder pain', *Occupational Medicine,* 62 (6), 451-54.

Berg-Beckhoff, G., Østergaard, H., and Jepsen, J. R. (2016), 'Prevalence and predictors of musculoskeletal pain among Danish fishermen - results from a cross-sectional survey', *Journal of Occupational Medicine and Toxicology,* 11 (1), 1-9.

Bernard, C., et al. (2011), 'Biomechanical and psychosocial work exposures and musculoskeletal symptoms among vineyard workers', *Journal of Occupational Health,* 53 (5), 297-311.

Bilberg, A., et al. (2014), 'Work status in patients with early rheumatoid arthritis: emphasis on shoulder function and mechanical exposure', *Scandinavian Journal of Rheumatology,* 43 (2), 119-23.

Björing, G. and Hägg, G. M. (2000), 'Musculoskeletal exposure of manual spray painting in the woodworking industry - An ergonomic study on painters', *International Journal of Industrial Ergonomics,* 26 (6), 603-14.

Björksten, M. G., et al. (2001), 'Reported neck and shoulder problems in female industrial workers: the importance of factors at work and at home', *International Journal of Industrial Ergonomics,* 27 (3), 159-70.

Björksten, M. G., et al. (1996), 'Neck and shoulder ailments in a group of female industrial workers with monotonous work', *Annals of Occupational Hygiene,* 40 (6), 661-73.

Bodin, J., et al. (2014), 'Natural course of rotator cuff syndrome in a French working population', *American Journal of Industrial Medicine,* 57 (6), 683-94.

Bonde, J. P., et al. (2003), 'Prognosis of shoulder tendonitis in repetitive work: a follow up study in a cohort of Danish industrial and service workers', *Occupational & Environmental Medicine,* 60 (9), E8.

Borstad, J., et al. (2009), 'A longitudinal analysis of the effects of a preventive exercise programme on the factors that predict shoulder pain in construction apprentices', *Ergonomics,* 52 (2), 232-44.

Bos, E., et al. (2007), 'Risk factors and musculoskeletal complaints in non-specialized nurses, IC nurses, operation room nurses, and X-ray technologists', *International Archives of Occupational & Environmental Health,* 80 (3), 198-206.

Bulduk, E. T., et al. (2014), 'Assessing exposure to risk factors for work-related musculoskeletal disorders using Quick Exposure Check (QEC) in taxi drivers', *International Journal of Industrial Ergonomics,* 44 (6), 817-20.

Carugno, M., et al. (2012), 'Physical and psychosocial risk factors for musculoskeletal disorders in Brazilian and Italian nurses', *Cadernos de Saude Publica,* 28 (9), 1632-42.

Cassou, B., et al. (2002), 'Chronic neck and shoulder pain, age, and working conditions: longitudinal results from a large random sample in France', *Occupational and Environmental Medicine,* 59 (8), 537-44.

Chiang, H. C., et al. (1993), 'Prevalence of shoulder and upper-limb disorders among workers in the fish-processing industry', *Scandinavian Journal of Work, Environment & Health,* 19 (2), 126-31.

Chowdhury, S. S., Boricha, J., and Yardi, S. (2012), 'Identification of awkward postures that cause discomfort to Liquid Petroleum Gas workers in Mumbai, India', *Indian Journal of Occupational and Environmental Medicine,* 16 (1), 3-8.

Chung, M. K., Lee, I., and Kee, D. (2005), 'Quantitative postural load assessment for whole body manual tasks based on perceived discomfort', *Ergonomics,* 48 (5), 492-505.

Coffin, C. T. (2014), 'Work-related musculoskeletal disorders in sonographers: A review of causes and types of injury and best practices for reducing injury risk', *Reports in Medical Imaging,* 7 (1), 15-26.

Crawford, J. O., et al. (2008), 'Musculoskeletal disorders within the telecommunications sector-A systematic review', *International Journal of Industrial Ergonomics,* 38 (1), 56-72.

Cromie, J. E., Robertson, V. J., and Best, M. O. (2000), 'Work-related musculoskeletal disorders in physical therapists: prevalence, severity, risks, and responses', *Physical Therapy,* 80 (4), 336-51.

Dalbøge, A., et al. (2016), 'Upper arm elevation and repetitive shoulder movements: a general population job exposure matrix based on expert ratings and technical measurements', *Occupational and Environmental Medicine,* 73 (8), 553-60.

Dehdashti, A., Mahjoubi, Z., and Salarinia, A. (2015), 'Impact of nurse's work related body postures on their musculoskeletal disorders. [Persian]', *Koomesh,* 16 (3), 338-46.

Duarte, F. and Serranheira, F. (2015), 'Dental hygienists self-reported work-related musculoskeletal disorders symptoms and task demands. [Portuguese]', *Revista Portuguesa de Saude Publica,* 33 (1), 49-56.

Ekberg, K., et al. (1995), 'Cross-sectional study of risk factors for symptoms in the neck and shoulder area', *Ergonomics,* 38 (5), 971.

Ekberg, K., et al. (1994), 'Case-control study of risk factors for disease in the neck and shoulder area', *Occupational and Environmental Medicine,* 51 (4), 262-66.

Engström, T., Hanse, J. J., and Kadefors, R. (1999), 'Musculoskeletal symptoms due to technical preconditions in long cycle time work in an automobile assembly plant: a study of prevalence and relation to psychosocial factors and physical exposure', *Applied Ergonomics,* 30 (5), 443-53.

Fagarasanu, M. and Kumar, S. (2003), 'Shoulder musculoskeletal disorders in industrial and office work', *Journal of Musculoskeletal Research,* 7 (1), 1-14.

Feveile, H., Jensen, C., and Burr, H. (2002), 'Risk factors for neck-shoulder and wrist-hand symptoms in a 5-year follow-up study of 3,990 employees in Denmark', *International Archives of Occupational and Environmental Health,* 75 (4), 243-51.

Finsen, L., Christensen, H., and Bakke, M. (1998), 'Musculoskeletal disorders among dentists and variation in dental work', *Applied Ergonomics,* 29 (2), 119-25.

Fredriksson, K., et al. (2005), 'Occupational chronic neck and shoulder pain: Study conducted in Sweden', *Occupational Ergonomics,* 5 (2), 79-88.

Fredriksson, K., et al. (2002), 'Work environment and neck and shoulder pain: the influence of exposure time. Results from a population based case-control study', *Occupational & Environmental Medicine,* 59 (3), 182-8.

Fredriksson, K., et al. (2001), 'The impact on musculoskeletal disorders of changing physical and psychosocial work environment conditions in the automobile industry', *International Journal of Industrial Ergonomics,* 28 (1), 31-45.

Fredriksson, K., et al. (2000), 'Risk factors for neck and shoulder disorders: A nested case-control study covering a 24-year period', *American Journal of Industrial Medicine,* 38 (5), 516-28.

Fredriksson, K., et al. (1999), 'Risk factors for neck and upper limb disorders: results from 24 years of follow up.[Erratum appears in Occup Environ Med 1999 May;56(5):358]', *Occupational & Environmental Medicine,* 56 (1), 59-66.

Frost, P. and Andersen, J. H. (1999), 'Shoulder impingement syndrome in relation to shoulder intensive work', *Occupational and Environmental Medicine,* 56 (7), 494-98.

Garg, A., Hegmann, K. T., and Kapellusch, J. (2006), 'Short-cycle overhead work and shoulder girdle muscle fatigue', *International Journal of Industrial Ergonomics,* 36 (6), 581-97.

Gerr, F., et al. (2014), 'A prospective study of musculoskeletal outcomes among manufacturing workers: I. Effects of physical risk factors', *Human Factors,* 56 (1), 112-30.

Geuskens, G. A., et al. (2011), 'A high physical workload and high job demands hamper the good prognosis in physical functioning in persons with early inflammatory joint conditions', *Rheumatology,* 50 (4), 789-98.

Grieco, A., et al. (1998), 'Epidemiology of musculoskeletal disorders due to biomechanical overload', *Ergonomics,* 41 (9), 1253-60.

Grooten, W. J., et al. (2007), 'The influence of work-related exposures on the prognosis of neck/shoulder pain', *European Spine Journal,* 16 (12), 2083-91.

Grooten, W. J. A., et al. (2004), 'Seeking Care for Neck/Shoulder Pain: A Prospective Study of Work-Related Risk Factors in a Healthy Population', *Journal of Occupational and Environmental Medicine,* 46 (2), 138-46.

Hanvold, T. N., et al. (2014), 'A longitudinal study on risk factors for neck and shoulder pain among young adults in the transition from technical school to working life', *Scandinavian Journal of Work, Environment & Health,* 40 (6), 597.

Hanvold, T. N., Wærsted, M., and Veiersted, K. B. (2012), 'Long periods with uninterrupted muscle activity related to neck and shoulder pain', *Work,* 41 Suppl 1, 2535-8.

Harcombe, H., et al. (2010), 'Physical and psychosocial risk factors for musculoskeletal disorders in New Zealand nurses, postal workers and office workers', *Injury Prevention,* 16 (2), 96-100.

Harkness, E. F., et al. (2004), 'Mechanical injury and psychosocial factors in the work place predict the onset of widespread body pain: a two-year prospective study among cohorts of newly employed workers', *Arthritis Rheum,* 50 (5), 1655-64.

Hayes, M. J., Taylor, J. A., and Smith, D. R. (2012), 'Predictors of work-related musculoskeletal disorders among dental hygienists', *International Journal of Dental Hygiene,* 10 (4), 265-9.

Henry, L. J., et al. (2015), 'Patterns of work-related musculoskeletal disorders among workers in palm plantation occupation', *Asia-Pacific Journal of Public Health,* 27 (2), NP1785-92.

Herin, F., et al. (2014), 'Predictive risk factors for chronic regional and multisite musculoskeletal pain: a 5-year prospective study in a working population', *Pain,* 155 (5), 937-43.

Herin, F., et al. (2012), 'Predictors of chronic shoulder pain after 5 years in a working population', *Pain,* 153 (11), 2253-9.

Holmström, E. B., Lindell, J., and Moritz, U. (1992), 'Low back and neck/shoulder pain in construction workers: Occupational workload and psychosocial risk factors. Part 2: Relationship to neck and shoulder pain', *Spine,* 17 (6), 672-77.

Holte, K. A. and Westgaard, R. H. (2002), 'Further studies of shoulder and neck pain and exposures in customer service work with low biomechanical demands', *Ergonomics,* 45 (13), 887.

Hooftman, W. E., et al. (2004), 'Gender differences in the relations between work-related physical and psychosocial risk factors and musculoskeletal complaints', *Scandinavian Journal of Work, Environment & Health,* 30 (4), 261-78.

Hoppmann, R. A. (2001), 'Instrumental musicians' hazards', *Occupational Medicine,* 16 (4), 619-31, iv-v.

Hughes, R. E., Silverstein, B. A., and Evanoff, B. A. (1997), 'Risk factors for work-related musculoskeletal disorders in an aluminum smelter', *American Journal of Industrial Medicine,* 32 (1), 66-75.

Hwang, U. J., et al. (2017), 'Predictors of upper trapezius pain with myofascial trigger points in food service workers: The STROBE study', *Medicine,* 96 (26), e7252.

Junejo, M. A., Tahir, S. M., and Behan, R. B. (2017), 'Prevalence and risk factors for work related musculoskeletal disorders among sonographer of Sindh Province Pakistan', *Journal of the Liaquat University of Medical and Health Sciences,* 16 (1), 29-36.

Kamwendo, K., Linton, S. J., and Moritz, U. (1991), 'Neck and shoulder disorders in medical secretaries. Part I. Pain prevalence and risk factors', *Scandinavian Journal of Rehabilitation Medicine,* 23 (3), 127-33.

Kamwendo, K., Linton, S. J., and Moritz, U. (1991), 'Neck and shoulder disorders in medical secretaries. Part II. Ergonomical work environment and symptom profile', *Scandinavian Journal of Rehabilitation Medicine,* 23 (3), 135-42.

Kao, H. W., et al. (2009), 'Work-related musculoskeletal disorders among medical staff in a radiology department', *Journal of Medical Sciences,* 29 (3), 119-24.

Kim, J. H., et al. (2015), 'Risk factors of work-related upper extremity musculoskeletal disorders in male cameramen', *Annals of Occupational and Environmental Medicine,* 27 (1) (no pagination) (5).

Koohpayehzadeh, J., et al. (2016), 'The role of work- related physical and psychological factors on prevalence of neck/shoulder complaints among nurses: A multicentric study', *Medical Journal of the Islamic Republic of Iran,* 30, 470.

Koshy, J. M., et al. (2016), 'A study to evaluate the prevalence of postural problems among the dentists in Chennai, Tamil Nadu, India', *Research Journal of Pharmaceutical, Biological and Chemical Sciences,* 7 (3), 2552-59.

Kumar, P., et al. (2016), 'Work-related pains among the workers associated with pineapple peeling in small fruit processing units of North East India', *International Journal of Industrial Ergonomics,* 53, 124-29.

Kumar, R. S., Manish, N. G., and Ferreira, A. M. (2000), 'Occupational hazards among dental surgeons', *Indian Journal of Occupational and Environmental Medicine,* 4 (3), 139-41.

Leijon, O., et al. (2007), 'Different working and living conditions and their associations with persistent neck/shoulder and/or low back disorders', *Occupational & Environmental Medicine,* 64 (2), 115-21.

Leijon, O., et al. (2006), 'Target groups for prevention of neck/shoulder and low back disorders: An exploratory cluster analysis of working and living conditions', *Work,* 27 (2), 189-204.

Lin, C. L., et al. (2010), 'Evaluation of perceived discomfort in repetitive arm reaching and holding tasks', *International Journal of Industrial Ergonomics,* 40 (1), 90.

Lin, J. D., et al. (2008), 'Perceived adverse occupational health effects in hospital personnel: An exploration of the effects of the workplace environment', *Journal of Medical Sciences,* 28 (6), 227-32.

Linaker, C. H. and Walker-Bone, K. (2015), 'Shoulder disorders and occupation', *Best Practice & Research in Clinical Rheumatology,* 29 (3), 405-23.

Long, J., et al. (2011), 'Risk factors for physical discomfort in Australian optometrists', *Optometry and Vision Science,* 88 (2), 317-26.

Lowe, B. D., et al. (2017), 'Evaluation of a Workplace Exercise Program for Control of Shoulder Disorders in Overhead Assembly Work', *Journal of Occupational and Environmental Medicine,* 59 (6), 563.

Lowry, V., et al. (2017), 'Efficacy of workplace interventions for shoulder pain: A systematic review and meta-analysis', *Journal of Rehabilitation Medicine,* 49 (7), 529-42.

Lu, J. M., Twu, L. J., and Wang, M. J. J. (2016), 'Risk assessments of work-related musculoskeletal disorders among the TFT-LCD manufacturing operators', *International Journal of Industrial Ergonomics,* 52, 40-51.

Madeleine, P., et al. (2012), 'Level of self-reported neck/shoulder pain and biomechanical workload in cleaners', *Work,* 41 Suppl 1, 447-52.

Maulik, S., et al. (2014), 'Evaluation of the working posture and prevalence of musculoskeletal symptoms among medical laboratory technicians', *Journal of Back & Musculoskeletal Rehabilitation,* 27 (4), 453-61.

McKinnon, C. D., et al. (2014), 'The effect of police cruiser restraint cage configuration on shoulder discomfort, muscular demands, upper limb postures, and task performance during simulated police patrol', *Applied Ergonomics,* 45 (6), 1414.

Memarpour, M., et al. (2013), 'Work-related musculoskeletal disorders among Iranian dentists', *Work,* 45 (4), 465-74.

Miranda, H., et al. (2008), 'Physical work and chronic shoulder disorder. Results of a prospective population-based study', *Annals of the Rheumatic Diseases,* 67 (2), 218-23.

Molteni, G., et al. (1996), '[Epidemiology of musculoskeletal disorders caused by biomechanical overload (WMSDs)]', *Medicina del Lavoro,* 87 (6), 469-81.

Moreira-Silva, I., et al. (2016), 'The Effects of Workplace Physical Activity Programs on Musculoskeletal Pain: A Systematic Review and Meta-Analysis', *Workplace Health & Safety,* 64 (5), 210-22.

Morse, T., et al. (2007), 'Musculoskeletal disorders of the neck and shoulder in dental hygienists and dental hygiene students', *Journal of Dental Hygiene,* 81 (1), 10.

Muggleton, J. M., Allen, R., and Chappell, P. H. (1999), 'Hand and arm injuries associated with repetitive manual work in industry: A review of disorders, risk factors and preventive measures', *Ergonomics,* 42 (5), 714-39.

Mukhopadhyay, P., O'Sullivan, L. W., and Gallwey, T. J. (2009), 'Upper limb discomfort profile due to intermittent isometric pronation torque at different postural combinations of the shoulder-arm system', *Ergonomics,* 52 (5), 584.

Mukhopadhyay, P., O'Sullivan, L. W., and Gallwey, T. J. (2007), 'Effects of upper arm articulations on shoulder-arm discomfort profile in a pronation task', *Occupational Ergonomics,* 7 (3), 169-81.

Mukhopadhyay, P., O'Sullivan, L. W., and Gallwey, T. J. (2007), 'Estimating upper limb discomfort level due to intermittent isometric pronation torque with various combinations of elbow angles, forearm rotation angles, force and frequency with upper arm at 90° abduction', *International Journal of Industrial Ergonomics,* 37 (4), 313-25.

Myers, D., Silverstein, B., and Nelson, N. A. (2002), 'Predictors of shoulder and back injuries in nursing home workers: A prospective study', *American Journal of Industrial Medicine,* 41 (6), 466-76.

Møller, S. P., et al. (2018), 'Risk of subacromial shoulder disorder in airport baggage handlers: combining duration and intensity of musculoskeletal shoulder loads', *Ergonomics,* 61 (4), 576-87.

Ng, Y. G., et al. (2015), 'Risk factors of musculoskeletal disorders among oil palm fruit harvesters during early harvesting stage', *Annals of Agricultural & Environmental Medicine,* 22 (2), 286-92.

Nicoletti, S., et al. (2008), '[Upper limb work-related musculoskeletal disorders (UL-WMSDs): a retrospective cohort study in three large factories of the upholstered furniture industry]', *Medicina del Lavoro,* 99 (4), 281-96.

Nicoletti, S., et al. (2008), '[Prevalence of upper limb work-related musculoskeletal disorders (UL-WMSDs) in workers of the upholstered furniture industry]', *Medicina del Lavoro,* 99 (4), 271-80.

Nordander, C., et al. (2009), 'Risk of musculoskeletal disorders among females and males in repetitive/constrained work', *Ergonomics,* 52 (10), 1226-39.

Nyman, T., et al. (2009), 'Physical workload, low back pain and neck-shoulder pain: a Swedish twin study', *Occupational & Environmental Medicine,* 66 (6), 395-401.

Nyman, T., et al. (2007), 'Work postures and neck-shoulder pain among orchestra musicians', *American Journal of Industrial Medicine,* 50 (5), 370-76.

Olsson, A. R., et al. (2004), 'Occupations and exposures in the work environment as determinants for rheumatoid arthritis', *Occupational & Environmental Medicine,* 61 (3), 233-8.

Ono, Y., et al. (2002), 'Associations of Length of Employment and Working Conditions with Neck, Shoulder and Arm Pain among Nursery School Teachers', *Industrial Health,* 40 (2), 149-58.

Palmer, K. T. (1996), 'Musculoskeletal problems in the tomato growing industry: "Tomato Trainer's Shoulder"?', *Occupational Medicine,* 46 (6), 428-31.

Park, J. K. (2016), 'Job Hazard Analyses for Musculoskeletal Disorder Risk Factors in Pressing Operations of Dry-cleaning Establishments', *Safety and Health at Work,* 7 (4), 389-93.

Park, M. H., Kim, H. G., and Cho, J. H. (2015), 'Risk Factors for Musculoskeletal Symptoms Among Korean Broadcast Actors', *Annals of Global Health,* 81 (4), 475-81.

Pehkonen, I., et al. (2009), 'Prospective study on shoulder symptoms among kitchen workers in relation to self-perceived and observed work load', *Occupational & Environmental Medicine,* 66 (6), 416-23.

Phelan, D. and O'Sullivan, L. (2014), 'Shoulder muscle loading and task performance for overhead work on ladders versus Mobile Elevated Work Platforms', *Applied Ergonomics,* 45 (6), 1384.

Pinar, R. (2010), 'Work-related musculoskeletal disorders in Turkish hospital nurses. [Turkish]', *Turkiye Klinikleri Journal of Medical Sciences,* 30 (6), 1869-75.

Pourmahabadian, M. and Azam, K. (2006), 'Evaluation of risk factors associated with work-related musculoskeletal disorders of upper limbs extremity among press workers', *Pakistan Journal of Medical Sciences,* 22 (4), 379-84.

Putz-Anderson, V. and Galinsky, T. L. (1993), 'Psychophysically determined work durations for limiting shoulder girdle fatigue from elevated manual work', *International Journal of Industrial Ergonomics,* 11 (1), 19-28.

Rahman, M. N., Rani, M. R., and Rohani, J. M. (2012), 'Investigation of work-related musculoskeletal disorders in wall plastering jobs within the construction industry', *Work,* 43 (4), 507-14.

Robstad Andersen, G. and Westgaard, R. H. (2014), 'Perceived occupational exposures of home care workers and the association to general tension, shoulder-neck and low back pain', *Work,* 49 (4), 723-33.

Roll, S. C., et al. (2012), 'An analysis of occupational factors related to shoulder discomfort in diagnostic medical sonographers and vascular technologists', *Work,* 42 (3), 355-65.

Roquelaure, Y., et al. (2006), 'Epidemiologic surveillance of upper-extremity musculoskeletal disorders in the working population', *Arthritis & Rheumatism,* 55 (5), 765-78.

Roquelaure, Y., et al. (2002), 'Active epidemiological surveillance of musculoskeletal disorders in a shoe factory', *Occupational & Environmental Medicine,* 59 (7), 452-8.

Ryu, T., et al. (2005), 'Postural Stress of Complex Shoulder Postures with External Loads Based on Perceived Discomfort', *Asian Journal of Ergonomics,* 6 (2).

Sabri, N. and Rampal, K. G. (2014), 'Prevalence and risk factors of musculoskeletal problems among cashiers in Kuala Lumpur and Selangor', *Archives of Pharmacy Practice,* 1), S2.

Sadeghian, F., et al. (2013), 'Predictors of incident and persistent neck/shoulder pain in Iranian workers: a cohort study', *PLoS ONE [Electronic Resource],* 8 (2), e57544.

Sakakibara, H., et al. (1993), '[Musculoskeletal symptoms and working postures in pear and apple orchard workers]', *Sangyo Igaku - Japanese Journal of Industrial Health,* 35 (6), 530-6.

Sansone, V. C., et al. (2015), 'Are occupational repetitive movements of the upper arm associated with rotator cuff calcific tendinopathies?', *Rheumatology International,* 35 (2), 273-80.

Scuffham, A. M., et al. (2010), 'Prevalence and risk factors associated with musculoskeletal discomfort in New Zealand veterinarians', *Applied Ergonomics,* 41 (3), 444-53.

Seitz, A. L., et al. (2011), 'Mechanisms of rotator cuff tendinopathy: intrinsic, extrinsic, or both?', *Clin Biomech (Bristol, Avon),* 26 (1), 1-12.

Silverstein, B. A., et al. (2006), 'Natural course of nontraumatic rotator cuff tendinitis and shoulder symptoms in a working population', *Scandinavian Journal of Work, Environment & Health,* 32 (2), 99-108.

Simsek, S., Senocak, O., and Kavlak, E. (2014), 'Neck and shoulder pains in medical secretaries. [Turkish, English]', *Fizyoterapi Rehabilitasyon,* 1), S33-S34.

Skov, T., Borg, V., and Orhede, E. (1996), 'Psychosocial and physical risk factors for musculoskeletal disorders of the neck, shoulders, and lower back in salespeople', *Occupational and Environmental Medicine,* 53 (5), 351-56.

Smedley, J., et al. (2003), 'Risk factors for incident neck and shoulder pain in hospital nurses', *Occupational & Environmental Medicine,* 60 (11), 864-9.

Snels, I. A. K., et al. (2002), 'Risk factors for hemiplegic shoulder pain: A systematic review', *Critical Reviews in Physical and Rehabilitation Medicine,* 14 (3-4), 223-33.

Solidaki, E., et al. (2010), 'Work-related and psychological determinants of multisite musculoskeletal pain', *Scandinavian Journal of Work, Environment and Health, Supplement,* 36 (1), 54-61.

Sommerich, C. M., McGlothlin, J. D., and Marras, W. S. (1993), 'Occupational risk factors associated with soft tissue disorders of the shoulder: A review of recent investigations in the literature', *Ergonomics,* 36 (6), 697-717.

Sood, D., Nussbaum, M. A., and Hager, K. (2007), 'Fatigue during prolonged intermittent overhead work: reliability of measures and effects of working height', *Ergonomics,* 50 (4), 497-513.

Stamou, M., et al. (2007), 'Work related musculoskeletal disorders among physical therapists in Greece', *Review of Clinical Pharmacology and Pharmacokinetics, International Edition,* 21 (3), 261-69.

Sterud, T., Johannessen, H. A., and Tynes, T. (2014), 'Work-related psychosocial and mechanical risk factors for neck/shoulder pain: a 3-year follow-up study of the general working population in Norway', *International Archives of Occupational and Environmental Health,* 87 (5), 471-81.

Stock, S. R. (1991), 'Workplace ergonomic factors and the development of musculoskeletal disorders of the neck and upper limbs: a meta-analysis', *American Journal of Industrial Medicine,* 19 (1), 87-107.

Sundstrup, E., et al. (2013), 'Participatory ergonomic intervention versus strength training on chronic pain and work disability in slaughterhouse workers: study protocol for a single-blind, randomized controlled trial', *BMC Musculoskeletal Disorders,* 14, 67.

Tornqvist, E. W., et al. (2001), 'The influence on seeking care because of neck and shoulder disorders from work-related exposures', *Epidemiology,* 12 (5), 537-45.

Truszczynska, A., Scherer, A., and Drzal-Grabiec, J. (2016), 'The occurrence of overload at work and musculoskeletal pain in young physiotherapists', *Work,* 54 (3), 609-16.

Tsigonia, A., et al. (2009), 'Musculoskeletal disorders among cosmetologists', *International Journal of Environmental Research & Public Health [Electronic Resource],* 6 (12), 2967-79.

Uddin, S., et al. (2016), 'To see the work related musculoskeletal disorders among college teachers', *Medical Forum Monthly,* 27 (6), 39-43.

Valachi, B. and Valachi, K. (2003), 'Mechanisms leading to musculoskeletal disorders in dentistry', *Journal of the American Dental Association,* 134 (10), 1344-50.

van der Molen, H. F., Sluiter, J. K., and Frings-Dresen, M. H. (2009), 'The use of ergonomic measures and musculoskeletal complaints among carpenters and pavers in a 4.5-year follow-up study', *Ergonomics,* 52 (8), 954-63.

van Reenen, H. H. H., et al. (2006), 'Is an imbalance between physical capacity and exposure to work-related physical factors associated with low-back, neck or shoulder pain?', *Scandinavian Journal of Work, Environment & Health,* 32 (3), 190-97.

Vasseljen, O., Holte, K. A., and Westgaard, R. H. (2001), 'Shoulder and neck complaints in customer relations: individual risk factors and perceived exposures at work', *Ergonomics,* 44 (4), 355-72.

Veiersted, K. B., et al. (2008), 'Effect of an intervention addressing working technique on the biomechanical load of the neck and shoulders among hairdressers', *Applied Ergonomics,* 39 (2), 183-90.

Viikari-Juntura, E. (2010), 'Increasing evidence of physical loads as risk factors for specific shoulder disorders', *Scandinavian Journal of Work, Environment and Health, Supplement,* 36 (3), 185-87.

Wang, P. C., et al. (2010), 'Follow-up of neck and shoulder pain among sewing machine operators: The Los Angeles garment study', *American Journal of Industrial Medicine,* 53 (4), 352-60.

Werner, R. A., et al. (2005), 'Predictors of upper extremity discomfort: a longitudinal study of industrial and clerical workers', *J Occup Rehabil,* 15 (1), 27-35.

Widanarko, B., et al. (2015), 'Interaction between physical and psychosocial risk factors on the presence of neck/shoulder symptoms and its consequences', *Ergonomics,* 58 (9), 1507-18.

Widanarko, B., et al. (2011), 'Prevalence of musculoskeletal symptoms in relation to gender, age, and occupational/industrial group', *International Journal of Industrial Ergonomics,* 41 (5), 561-72.

Woldendorp, K. H., et al. (2016), 'No association between posture and musculoskeletal complaints in a professional bassist sample', *European Journal of Pain,* 20 (3), 399-407.

Yue, P., Liu, F., and Li, L. (2012), 'Neck/shoulder pain and low back pain among school teachers in China, prevalence and risk factors', *BMC Public Health,* 12 (1), 789.

Östergren, P. O., et al. (2005), 'Incidence of shoulder and neck pain in a working population: effect modification between mechanical and psychosocial exposures at work? Results from a one year follow up of the Malmö shoulder and neck study cohort', *Journal of Epidemiology & Community Health,* 59 (9), 721-8.
