# Supplementary material for: Work above shoulder level and shoulder complaints: a systematic review
Source: Int Arch Occup Environ Health. 2020 Jun 22;93(8):925–54. doi: 10.1007/s00420-020-01551-4 (PMC7519900; doi:10.1007/s00420-020-01551-4)
Supplement: Supplementary file 3 — Supplementary file3 (DOCX 66 kb) [file 420_2020_1551_MOESM3_ESM.docx]

Appendix 3: Quality assessment

The scoring scheme for quality assessment included items on Study population (part A), Exposure measurements (part B), Outcome measurement (part C), Analysis and data presentation (part D) and Intervention (part E – only applied for intervention studies). Below the items from parts A-D are reproduced together with nine examples on scoring of included papers: all three included papers with case-control design, and three papers each from prospective cohort studies and cross-sectional studies. No intervention studies were included in the present review, and part E of the scoring scheme is not shown. Not all items were relevant for all study designs, as can been seen from the examples below.

Case-control studies **1**: Dalbøge 2017 **2**: Punnett 2000 **3**: Seidler 2011

Prospective cohort studies **4**: Bodin 2012a **5**: Bovenzi 2015 **6**: Harkness 2003

Cross-sectional studies **7**: Silverstein 2008 **8**: Sim 2006 **9**: Svendsen 2004b

| **A** | **Study population:** | **1** | **2** | **3** | **4** | **5** | **6** | **7** | **8** | **9** |
| --- | --- | --- | --- | --- | --- | --- | --- | --- | --- | --- |
| 1 | Positive if the main feature (type of work, description of sampling frame, and distribution by age and gender) of the study population were stated: ***1*** | **1** | **1** | **1** | **1** | **1** | **1** | **1** | **1** | **1** |
| 2 | External validity: Positive if study population is representative for a defined working population (for a defined type of work, for a defined branch, or for any type of work, etc): *Specific inclusion criteria for a defined working population:* ***1*** | **1** | **1** | **1** | **1** | **0** | **1** | **1** | **1** | **1** |
| 3 | External validity: Positive if study population is representative for a defined working population (for a defined type of work, for a defined branch, or for any type of work, etc): *Subjects recruited from >2 organizations/units:* ***2****; Subjects recruited from 2 organizations / units:* ***1*** |  |  |  | **2** | **2** | **2** | **2** | **2** | **2** |
| 4 | Selection bias: Positive if possible problems with selection bias in recruitment are addressed and avoided? *Yes:* ***2****; No obvious major recruitment problems:* ***1****; No information:* ***0*** | **2** | **1** | **1** | **1** | **2** | **1** | **0** | **2** | **1** |
| 5 | Selection bias: Positive if exclusion criteria are specific and do not allow selection bias: ***1*** | **1** | **1** | **1** | **1** | **1** | **1** | **1** | **0** | **1** |
| 6 | Positive if the participation rate at the beginning of the study was adequate: | **1** | **3** | **1** | **3** | **3** | **3** | **1** | **1** | **1** |
|  | *≥85%:* ***3****; 75-84%:* ***2****; 50-74%:* ***1****; < 50%:* ***0*** |  |  |  |  |  |  |  |  |  |
| 7 | Positive if the response at follow-up was adequate: |  |  |  | **2** | **1** | **3** |  |  |  |
|  | *≥85% of included subjects:* ***3****; 75–84%:* ***2****; 50–74%:* ***1****, <50%:* ***0*** |  |  |  |  |  |  |  |  |  |
| 8 | Sample size: *≥500:* ***2****; 50–499:* ***1****; <50:* ***0*** |  |  |  | **2** | **1** | **2** | **2** | **2** | **1** |
| 9 | Sample size, number of cases: *≥50:* ***2****; 25–49:* ***1****; <25:* ***0*** | **2** | **2** | **3** |  |  |  |  |  |  |
| 10 | Positive if subjects with chronic musculoskeletal complaints (>90 days) are excluded from the controls: ***1*** | **0** | **1** | **0** |  |  |  |  |  |  |
| 11 | Positive if the cases and referents were drawn from the same population: ***1*** | **1** | **1** | **1** |  |  |  |  |  |  |
| 12 | Positive if a clear definition of the cases and referents was stated: ***1*** | **1** | **1** | **1** |  |  |  |  |  |  |
| **A**  **A**  **A**  **A**  **A**  **A** | **Max obtainable score A** |  | **13** |  |  | **15** |  |  | **12** |  |
|  | **Received score A** | **10** | **12** | **9** | **13** | **11** | **14** | **8** | **9** | **8** |
|  | **Received score in percentage of max obtainable score A** | **77** | **92** | **69** | **87** | **73** | **93** | **67** | **75** | **67** |

**Continued …**

| **B** | **Exposure measurements:** | **1** | **2** | **3** | **4** | **5** | **6** | **7** | **8** | **9** |
| --- | --- | --- | --- | --- | --- | --- | --- | --- | --- | --- |
| 1 | Positive if level of physical exposure at work was measured for use in the analyses? *By measurements, objective observation or records:* ***2****; By questionnaires:* ***1****; Not assessed:* ***0***. | **2** | **2** | **1** | **1** | **2** | **1** | **2** | **1** | **2** |
| 2 | Positive if daily physical exposure *intensity* was measured? *By objective recordings:* ***2****; Assessed (using explicitly described methods of acceptable quality):* ***1****; Not assessed:* ***0***. | **2** | **2** | **0** | **0** | **2** | **1** | **2** | **0** | **2** |
| 3 | Positive if daily physical exposure *duration* was measured? *By objective recordings:* ***2****; Assessed (using explicitly described methods of acceptable quality):* ***1****; Not assessed:* ***0***. | **2** | **2** | **1** | **1** | **2** | **1** | **2** | **0** | **2** |
| 4 | Was duration of occupation in physically demanding work reported? *Yes:* ***1****; No:* ***0****.* | **1** | **1** | **1** | **0** | **1** | **1** | **1** | **1** | **1** |
| 5 | Positive if higher level of measurement scale for physical exposure were used in the analyses: *Interval or ratio scale:* ***3****; Rank scale (f. ex. high, medium, low):* ***2****; Dichotomous scale:* ***1****; Not quantified:* ***0*** | **2** | **3** | **2** | **1** | **3** | **2** | **3** | **1** | **3** |
| 6 | Positive if the assessed psychosocial/organizational exposure factors were explicitly defined: ***1*** | **1** | **0** | **0** | **1** | **1** | **1** | **1** | **1** | **0** |
| 7 | Positive if psychosocial/organizational exposures were assessed several times: >3 times: 3; 3 times: 2; 2 times: 1 | **0** | **0** | **0** | **0** | **0** | **2** |  |  |  |
| 8 | Positive if psychosocial/organizational exposures were assessed by objective observation or records and used in the analysis: **1** | **0** | **0** | **0** | **0** | **0** | **0** | **0** | **0** | **0** |
| 9 | Positive if psychosocial/organizational exposures were assessed by instruments that have been tested for validity and reliability (reference to psychometric data are given): ***1*** | **1** | **0** | **0** | **1** | **1** | **1** | **1** | **0** | **0** |
| 10 | Positive if higher level of measurement scale for psychosocial/organizational exposure were used in the analyses: *Interval or ratio scale:* ***3****; Rank scale (f. ex. high, medium, low):* ***2****; Dichotomous scale:* ***1;*** *Not quantified:* ***0*** | **2** | **0** | **0** | **1** | **2** | **1** | **1** | **1** | **0** |
| 11 | Positive if data on physical factors during leisure time were used in the analysis: ***1*** | **1** | **1** | **1** | **0** | **0** | **0** | **1** | **0** | **1** |
| 12 | Positive if data on psychosocial factors during leisure time were used in the analysis: ***1*** | **0** | **0** | **0** | **0** | **0** | **0** | **0** | **0** | **0** |
| 13 | Positive if data on historical exposures at work were used in the analysis: ***1*** | **1** | **0** | **0** | **0** | **1** | **0** | **0** | **0** | **1** |
| 14 | Positive if exposure assessment was blinded with respect to any symptoms or diseases: ***1*** | **1** | **1** | **1** | **0** | **1** | **0** | **0** | **0** | **1** |
| 15 | Positive if exposure was measured in an identical way among the cases and referents: ***1*** | **1** | **1** | **1** |  |  |  |  |  |  |
| **B**  **B** | **Max obtainable score B** |  | **24** |  |  | **23** |  |  | **20** |  |
|  | **Received score B** | **17** | **13** | **8** | **6** | **16** | **11** | **14** | **5** | **13** |
|  | **Received score in percentage of max obtainable score B** | **71** | **54** | **33** | **26** | **70** | **48** | **70** | **25** | **65** |

**Continued …**

| **C** | **Outcome measurement:** | **1** | **2** | **3** | **4** | **5** | **6** | **7** | **8** | **9** |
| --- | --- | --- | --- | --- | --- | --- | --- | --- | --- | --- |
| 1 | Positive if there is a definition of criteria for outcome (case definition): ***1*** | **1** | **1** | **1** | **1** | **1** | **1** | **1** | **1** | **1** |
| 2 | Positive if follow-up period is adequate: |  |  |  | **3** | **3** | **3** |  |  |  |
|  | *>1 yr:* ***3****; 7–12 months:* ***2****; 3–6 months:* ***1****; <3 months:* ***0*** |  |  |  |  |  |  |  |  |  |
| 3 | Positive if outcome was measured several times for each subject:  *>3 outcome meas­ure­ment times:* ***3****; 3 outcome measurement*  *times:* ***2****; 2 outcome measurement times:* ***1****; 1 sample time point:* ***0****.* |  |  |  | **1** | **2** | **2** |  |  |  |
| 4 | Positive if there is clinical diagnosis based on physical examination: ***1*** | **1** | **1** | **1** | **1** | **0** | **0** | **1** | **0** | **1** |
| 5 | Positive if physical examination is blinded to exposure status: ***1*** | **0** | **0** | **1** | **0** | **0** | **0** | **0** | **0** | **1** |
| 6 | Positive if data on outcome were collected using explicitly described methods of acceptable quality: ***1*** | **1** | **1** | **1** | **1** | **1** | **1** | **1** | **1** | **1** |
| 7 | Positive if incident cases were used (prospective enrolment): ***1*** | **0** | **0** | **0** |  |  |  |  |  |  |
| **C**  **C** | **Max obtainable score C** |  | **5** |  |  | **10** |  |  | **4** |  |
|  | **Received score C** | **3** | **3** | **4** | **7** | **7** | **7** | **3** | **2** | **4** |
|  | **Received score in percentage of max obtainable score C** | **60** | **60** | **80** | **70** | **70** | **70** | **75** | **50** | **100** |
| **D** | **Analysis and data presentation:** | **1** | **2** | **3** | **4** | **5** | **6** | **7** | **8** | **9** |
| 1 | Positive if the statistical models used were appropriate for the outcome studied and the measurement of the association estimated with the models were presented (including confidence intervals): ***1*** | **1** | **1** | **1** | **1** | **1** | **1** | **1** | **1** | **1** |
| 2 | Positive if the study controlled for confounding factors: | **3** | **2** | **2** | **2** | **3** | **3** | **2** | **2** | **2** |
|  | ***1 point for each of*** *the following factors:* ***Age, Gender, Education****.* |  |  |  |  |  |  |  |  |  |
| 3 | Positive if the study controlled for confounding factors: ***1 point*** *if at least two of the following factors were controlled for:* ***Smoking, Overweight, Physical activity****.* | **1** | **0** | **1** | **0** | **1** | **0** | **0** | **0** | **1** |
| 4 | Positive if the analyses controlled for history of the musculoskeletal disorder studied: ***2*** | **2** | **2** | **0** | **0** | **0** | **0** | **0** | **0** | **0** |
| 5 | Positive if the analyses controlled for history of other health problems: ***1*** | **1** | **1** | **0** | **0** | **0** | **0** | **0** | **0** | **0** |
| 6 | Positive if the number of cases in the multivariate analysis was at least 10 times the number of independent variables in the analysis: ***1*** | **1** | **1** | **1** | **1** | **1** | **1** | **1** | **1** | **1** |
| **D**  **D** | **Max obtainable score D** |  | **9** |  |  | **9** |  |  | **9** |  |
|  | **Received score D** | **9** | **7** | **5** | **4** | **6** | **5** | **4** | **4** | **5** |
|  | **Received score in percentage of max obtainable score D** | **100** | **78** | **56** | **44** | **67** | **56** | **44** | **44** | **56** |
| **SUMMARY**  **SUMMARY**  **SUMMARY**  **SUMMARY** | **Max obtainable score A-D** |  | **51** |  |  | **57** |  |  | **45** |  |
|  | **Total received score A-D** | **39** | **35** | **26** | **30** | **40** | **37** | **29** | **20** | **30** |
|  | **Total received score A-D in % of max obtainable score** | **76** | **69** | **51** | **53** | **70** | **65** | **64** | **44** | **67** |
