# Supplementary material for: Work above shoulder level and shoulder complaints: a systematic review
Source: Int Arch Occup Environ Health. 2020 Jun 22;93(8):925–54. doi: 10.1007/s00420-020-01551-4 (PMC7519900; doi:10.1007/s00420-020-01551-4)
Supplement: Supplementary file 4 — Supplementary file4 (DOCX 18 kb) [file 420_2020_1551_MOESM4_ESM.docx]

Appendix 4. Confounders included in the multivariate analyses

The table gives an overview of the variables included as confounders in multivariate analyses in the articles. The list does not take into account that some of the articles have considered other variables, but excluded them from the analysis because they did not show any relation in univariate analyses or because they strongly correlated with other variables included as confounders in the analyses. To get an overview of such conditions, the individual article must be studied. For articles that have stratified the analysis for gender or for specific age groups, or where only men or women are included in the survey, the table states that the analysis controls for age and gender.

| **Article** | **Confounders** |
| --- | --- |
| Bodin  2012a | Age, gender, BMI, temporary employment, high perceived physical exertion, low decision latitude |
| Bodin  2012b | Age, gender, work pace, repetitiveness of tasks, physical demands, exposure to cold temperatures, high psychological demands, low skill discretion, low supervisor support |
| Bodin  2012c | Age, gender, high perceived physical exertion, low coworker support, work with temporary workers |
| Bovenzi  2015 | Age, gender, BMI, smoking, drinking, education, physical activity, previous exposures to whole-body vibration and/or heavy workload, survey time |
| Coenen  2016 | Age, gender, external force exertion at the hands, body height and weight, number of years in the job |
| Dalbøge  2018a | Age, gender, region of residence, calendar year at start of follow-up, number of the particular follow-up year, repetition, hand-arm vibration |
| Dalbøge  2017 | Age, gender, BMI, smoking, leisure time shoulder intensive sports, diabetes mellitus, psychosocial strain, social support, demand, control, region of residence |
| Dalbøge  2014 | Age, gender, region of residence, calendar year at start of follow-up, number of the particular follow-up year |
| Descatha  2012 | Age, gender, BMI, regular sports activity, smoking, history of shoulder trauma, date of retirement |
| Engholm  2005 | Age, gender, vibration, lifting heavy burdens, stooping or twisted posture, poor information about future plans, lack of work-task variation, job responsibility too heavy, work health hazard anxiety, work psychological demanding, hurrying without reason, sleeping problems, depression |
| Hanvold  2015 | Age, gender, time, prior shoulder pain, self-reported mechanical workload, work demands, tobacco use, physical activity during leisure time |
| Harkness  2003 | Age, gender, occupation, lifting with on or two hands, pushing/pulling, monotonous work, other pain |
| Hoe  2012 | Age, gender, pain in other sites |
| Hooftman  2009 | Age, gender, education, nationality, years of employment, work hours, workdays, physical exposure, and psychosocial exposure were considered, however it is not clear what variables were included in the final model |
| Hoozemans  2002 | Age, gender, education, skill discretion, psychosocial work demands, coworker support, supervisor support |
| Koch  2017 | Age, gender, BMI, working sector, social climate, quantitative job demands, decision control, pacing control, psychological complaint severity Index, arm inclination during leisure |
| Leclerc  2004 | Age, gender, depressive symptoms, hit, bending forward, use of vibration tool, job control |
| Luime  2004a | Age, gender, The variable ‘hands above shoulder level’ did not remain in the multivariate models |
| Melchior  2006 | Age, gender, obesity, diabetes, thyroid disease, arthritis, repetitive movements, force exertion, hand behind trunk posture, arms away from body posture |
| Miranda 2005 | Age, gender, length of education, diabetes mellitus, duration of heavy lifting |
| Miranda 2001 | Age, gender, BMI, physical strenuous work, working with the trunk flexed forward, mental stress, jogging, dancing |
| Nahit  2001 | Age, gender |
| Niedhammer  1998 | Age, gender, psychological well-being, duration of employment, wage dependence on efficiency, stooping, holding heavy loads in position, checkout system |
| Nordander  2016 | Age, gender, exposure time, physical and psychosocial exposures |
| Punnett  2000 | Age, gender, length of employment, past injury, history of joint disease, recreational activities |
| Roquelaure 2011 | Age, gender, BMI, diabetes mellitus, high repetitiveness of tasks, high perceived workload, high psychological demands, low skill discretion, low decision authority |
| Seidler  2011 | Age, gender, place of residence, sports, lifting/carrying heavy loads, use of handheld vibrating tools |
| Silverstein  2009 | Age, gender, BMI |
| Silverstein  2008 | Age, gender, BMI |
| Sim  2006 | Age, gender, deprivation, work tasks, psychosocial factors, pottery work as main job |
| Smith  2009 | Age, gender, race, neck, elbow or hand/wrist symptoms at baseline, demand - control quadrants |
| Svendsen  2013 | Age, gender, BMI, job demands, job control, social support at work, smoking, |
| Svendsen  2004a | Age, gender, smoking, social support, job control, job demands |
| Svendsen  2004b | Age, gender, BMI, height, smoking, shoulder intensive sports, social support, job control, job demands |
